# Simulating the Epidemiological and Economic Impact of Paratuberculosis Control Actions in Dairy Cattle

**DOI:** 10.3389/fvets.2016.00090

**Published:** 2016-10-10

**Authors:** Carsten Kirkeby, Kaare Græsbøll, Søren Saxmose Nielsen, Lasse E. Christiansen, Nils Toft, Erik Rattenborg, Tariq Halasa

**Affiliations:** ^1^DTU VET, Section for Epidemiology, Technical University of Denmark, Frederiksberg, Denmark; ^2^DTU Compute, Section for Dynamical Systems, Department of Applied Mathematics and Computer Science, Technical University of Denmark, Frederiksberg, Denmark; ^3^Section for Animal Welfare and Disease Control, Department of Large Animal Sciences, University of Copenhagen, Frederiksberg, Denmark; ^4^SEGES, Agro Food Park, Aarhus, Denmark

**Keywords:** bioeconomic model, dairy cow, MAP, paratuberculosis, simulation model

## Abstract

We describe a new mechanistic bioeconomic model for simulating the spread of *Mycobacterium avium* subsp. *paratuberculosis* (MAP) within a dairy cattle herd. The model includes age-dependent susceptibility for infection; age-dependent sensitivity for detection; environmental MAP build up in five separate areas of the farm; *in utero* infection; infection *via* colostrum and waste milk, and it allows for realistic culling (i.e., due to other diseases) by including a ranking system. We calibrated the model using a unique dataset from Denmark, including 102 random farms with no control actions against spread of MAP. Likewise, four control actions recommended in the Danish MAP control program were implemented in the model based on reported management strategies in Danish dairy herds in a MAP control scheme. We tested the model parameterization in a sensitivity analysis. We show that a test-and-cull strategy is on average the most cost-effective solution to decrease the prevalence and increase the total net revenue on a farm with low hygiene, but not more profitable than no control strategy on a farm with average hygiene. Although it is possible to eradicate MAP from the farm by implementing all four control actions from the Danish MAP control program, it was not economically attractive since the expenses for the control actions outweigh the benefits. Furthermore, the three most popular control actions against the spread of MAP on the farm were found to be costly and inefficient in lowering the prevalence when used independently.

## Introduction

Paratuberculosis is a chronic infection in ruminants caused by *Mycobacterium avium* subsp. *paratuberculosis* (MAP), and resulting in financial losses to the dairy industry worldwide (1), where the prevalence of infected farms is believed to be substantial (2). Infected cattle can be subclinically infected for years until the animals develop acute diarrhea and eventually die. Infected animals also exhibit a decline in milk production. The annual economic loss due to MAP infection has been estimated to be as high as $200 million in the US alone (3). In Denmark, a national voluntary MAP control program was initiated in 2006, and in 2013, the estimated median true between- and within-herd prevalences among 925 herds participating in the control program were estimated to be 77 and 7%, respectively (4).

Simulation models have been used in evaluating the impact of different actions on the prevalence and spread of MAP in dairy herds [e.g., Ref. (5–8)]. These models predict that the within-herd true prevalence increases from 50 to 90% if no control actions are implemented (6–11). However, the within-herd true prevalence on farms in Denmark is much lower, around 7% (4), indicating an endemic situation with a stable prevalence. The previous models (mentioned above) are frequency-dependent models, in which the probability of infection depends directly on the number of infectious animals. Such models are suited for simulating epidemic situations [as described by Ryder et al. (12)]. Nevertheless, paratuberculosis is a slow progressing disease of endemic nature (7) and, hence, the chosen simulation model should reflect this nature. Therefore, we chose a density-dependent model, in which the probability of infection is dependent on the density of MAP in the herd. This model is suitable for modeling disease spread in endemic situations, especially when pathogens are spread through the environment (12). The objective of our study was to build a bioeconomic model framework, PTB-iCull calibrated to field data. Here, we describe the model and show how we used it to estimate the economic and epidemiologic impact of recommended MAP control actions from Denmark’s paratuberculosis control program.

## Materials and Methods

The PTB-iCull model is a stochastic, mechanistic, and dynamic discrete event simulation model that deals with the spread of MAP within a dairy herd in Denmark. It is written in R (13), and the current version of the model simulates a closed herd (without purchase of livestock) with a constricted herd size. The model consists of two main components: a herd dynamics component (LifeStep component) and a disease dynamics component. The time periods determining the life stage for each animal and the durations of the disease states of MAP infection are stochastically drawn from relevant distributions (Tables S1 and S2 in Supplementary Material). We used a dataset from the Danish Cattle Database hosted by SEGES (www.seges.dk) including milk records from 293,929 individual cows on 610 farms, recorded between year 2000 and 2013. In total, almost 5 million records were used to parameterize the cows in the model with regard to milk production and somatic cell count (SCC). We also used another dataset comprising 102 randomly chosen herds that were not enrolled in the Danish MAP control program. These herds were tested in August 2011 due to a sampling error where all milk-recorded herds were tested instead of just those in the control program. The error was detected after 5 days, so this cohort was considered as a random selection of non-program herds (program herds were excluded). All lactating animals in the herds were tested using the ID Screen (IDvet, Graebels, France) ELISA for detection of MAP (see also Section “ELISA” for test characteristics).

The simulation process is as follows: first, an initial herd is generated. The proportions of heifers, milking cows, and all other life steps are chosen to create a stable model, with regard to the number of animals in each life stage (Table S3 in Supplementary Material). In this study, the model represents a closed system (no purchase of animals), but it is possible to simulate an open herd. For each time step (1 day), the model tracks and updates the age of all animals in the herd, days in milk (DIM, the number of days a cow has been milking in the current lactation), the number of days that remain in the present life stage of each animal, and the number of days each animal has spent in the present disease state. For each day, we calculated the animal units in the herd and the number of slaughtered animals in each disease state.

We here use the model to simulate different scenarios. In each scenario, the farmer use a different strategy to control MAP on the farm, from no control to implementation of three control actions, and a test-and-cull strategy.

### Herd Dynamics

The model simulated a herd where the animals are kept indoors throughout the year. The cattle pass successively through the life stages in the model: calf; heifer; inseminated heifer; pregnant heifer; early lactation stage (after calving); inseminated cow; pregnant cow; and dry cow, and then again to the early lactation stage of next parity and so on. An animal can be culled at any stage of its life, which is modeled based on distributions in the Danish cattle population (Table S3 in Supplementary Material). We used the initial number of lactating cows as the maximum number of lactating cows during the simulated period. A typical Danish farm is divided into five sections based on the life stage of the animal. In the simulation model, animals are placed in one of the five farm sections: calves (0–1 year old), heifers (1 year old until first calving), lactating cows, dry cows, and calving pens. This reflects a common structure of farms in Denmark and allows us to simulate the spread of MAP within each section.

#### Insemination

When a heifer or a cow is inseminated, the insemination success (and hence the probability of continuing into pregnancy) is given by the probability of detecting the heat and the probability of conception following insemination. Of the unsuccessful inseminations, 90% (default) will wait 41 days before another insemination is attempted. The remaining 10% (default) will only wait one estrous cycle (21 days) before a new attempt, corresponding to the proportion of cows failing to conceive from an insemination. The default maximum number of insemination attempts before a cow is culled is seven (expert opinion).

#### Pregnancy and Calving

When an animal conceives, the number of days for pregnancy is drawn from a normal distribution (Table S1 in Supplementary Material). During the last stage of pregnancy, a cow is given a number of days in the dry period (Table S1 in Supplementary Material). Half of the calves are bull calves and are sold from the farm at a given price [161€ per calf (14)], and 4% of the calves are stillborn (Table S4 in Supplementary Material). Female calves proceed in the herd and are raised to heifers. After calving, the dam enters the early lactation stage where it produces milk. The number of days spent in the early lactation stage is drawn from a normal distribution (Table S1 in Supplementary Material). After the early milking stage, the cows are inseminated.

#### Culling

All heifers are inseminated, calve, and are put in the milking section. If there are more than 200 cows in the milking section, the excess number will be culled once per week. Culling is divided into two parts: voluntary and involuntary culling. Involuntary culling includes animals that are injured or subjected to other diseases and therefore sent to slaughter. These are randomly selected, but the probability of culling is dependent on the parity in order to balance the demographic structure of the herd. Data on the reasons for culling show that about 33% of cases are voluntary (Kasper Krogh, SEGES, personal communication, 2014). Voluntary culling in the model is carried out by prioritizing which cows should be culled, based on the information about simulated milk production, reproduction status, SCC, and repeated MAP ELISA values. We simulate quarterly observations of the milk production and SCC level for each cow. Practically, this is done by updating a cow-specific indicator measure for milk yield, and another cow-specific indicator measure for SCC, for each cow every time, there is a new observation. The observed level of milk or SCC for each cow is a weighted measure with 35% weight on the latest measurement and 65% on the previous value of the indicator. This results in an exponential smoothing mechanism where the four latest measurements account for 73% of the information, to mimic a farmer’s decision. Furthermore, the farmer can use flags to mark and prioritize cows for culling as in the national Danish Cattle Database (SEGES, Aarhus, Denmark). A cow is flagged each time, it exceeds a specified value for each of four categories: (1) milk yield is in the lowest 20% of cows on the farm; (2) number of insemination attempts in the current lactation is seven (default) or more; (3) observed SCC is above 200,000/ml (default), and (4) if test-and-cull strategy is used, a minimum of two of the last four MAP ELISA values are positive (according to the Danish MAP control program). Cull rates for each parity estimated from the dataset from SEGES (parity 1: 26%, parity 2: 40%, parity 3: 51%, parity 4: 59%, parity 5: 65%, and parity 6: 70%) are then added numerically to the number of flags per cow to balance the cullings. We kept the income from a culled cow fixed in the model at 483€ [600 kg × 0.805€ as listed in Kudahl et al. (14)]. Besides culling, cows can die due to background mortality at a cost of 79€ per carcass (from the rendering plant “DAKA SecAnim” 2014, see Table S4 in Supplementary Material).

#### Milk Yield

The model simulates a non-quota system without any assumptions about financial support or delivery contracts. Milk yield is recorded in kilograms of ECM (energy-corrected milk yield). We assigned an individual milk production level to each cow, relative to the other cows on the same farm. From this individual milk production level, we modeled the daily milk yield with two cow-specific parameters using the Wood lactation curve (15). We used a daily variation (SD) in the milk yield of 0.1.

Heifers inherit the milk production level from their dam. Animals get a new shape parameter for each parity. The shape parameter, *S*, is drawn from an exponential distribution:

(1) $$S∼\text{exp}\left(\lambda \right)$$

where λ is 6.735207. The individual milk level, α^M^, for a cow will be inherited by its offspring, with a regression tendency toward the mean:

(2) $$\alpha^{\text{M}}=N\left(1+\left(\alpha_{\text{dam}}^{\text{M}}-1\right)\cdot 0.13,\;0.19\right)$$

where α^M^ is the milk level of the dam, and 0.19 is the SD. We calculated DIM for each cow (and heifer) from when they have calved to when they are dried off. The farmer discards the milk in the first 2 days of each lactation period.

#### Somatic Cell Count

We modeled the SCC per cow during each lactation period. The values are generally inversely proportional to the milk yield over a lactation and have been parameterized by fitting a Wilmink style curve (16) to SCC data from the large dataset. The SCC level, α^C^, for each animal is drawn from a normal distribution as estimated from the dataset of Danish dairy herds:

(3) $$\alpha^{\text{C}}=N\left(1,\;0.051\right)$$

where 0.051 is the cow-specific variation estimated from the data. The SCC for the bulk tank milk is calculated for each day using a daily variation (SD) of 0.043, which is normally distributed before the log–log transformation.

We simulated a milk price according to the rules of Arla Foods (17). If the farmer has a bulk tank milk SCC count lower than 200,000 ml^−1^, the milk price increases by 2%. If the bulk tank milk SCC count is between 201,000 and 300,000 ml^−1^, the price increases by 1%. If the bulk tank milk SCC count is between 401,000 and 500,000 ml^−1^, the price decreases by 4%, and if the bulk tank milk SCC count is higher than 501,000 ml^−1^, the price decreases by 10%.

#### Feeding

We calculated the cost of feed for every simulated day. Farmers each have a specific feeding strategy, and therefore in order to include a standardized procedure in this model, we simulated a basic scenario for the cost of feed (Table S1 in Supplementary Material). For calves, the feeding costs are a linear function from 0€ at day 1 to the daily heifer costs when they are 1 year old, resulting in feeding cost for a calf of 170€ for their first year of life. For heifers and dry cows, we set the feed to cost 0.931€ per day, adding up to 340€ per year. The feed costs for raising a 2-year-old heifer are thus 510€ (170€ + 340€). For milking cows, we set the feed to cost 0.195€ per kilogram of milk produced per day (18).

### Disease Dynamics

Infected animals go through the following states of the disease in succession: susceptible, low-shedding, high-shedding, and affected. The low-shedding state corresponds to a stage where the animal is infected, asymptomatic, and without detectable levels of MAP-specific IgG1, whereas the high-shedding state corresponds to the infection stage where the cow becomes less able to control the infection, with increasing amounts of MAP-specific IgG1 and an increase in excretion of MAP (19). To scale the amount of MAP shed in the low-shedding, high-shedding, and affected states, we set the shedding amount to between 0 and 100% of the possible shedding amount. Thus, low-shedders shed 5%, high-shedders shed 20%, and affected cows shed 100% of the possible amount (see Table S2 in Supplementary Material). Once a cow begins to show clinical signs such as reduced milk yield or diarrhea, it transfers to the affected state. The number of days spent in each disease stage is drawn from a specified distribution and assigned to each animal (Table S2 in Supplementary Material). Susceptible animals can be infected with MAP from the environment. MAP is shed in the manure of infected animals, and viable MAP bacteria, once introduced to a farm section, are capable of persisting in the environment here. In the model, we keep track of every cow, the amount of MAP it sheds, and in which farm section. We, therefore, calculated the bacterial load in each farm section per day, and the daily survival rate for MAP was modeled by:

(4) $$\mathrm{Surv}\left(\mathrm{day}\right)=\exp\left(\mathrm{day}\cdot \left(\frac{\log\left(0.01\right)}{385}\right)\right)$$

where *day* is the number of days that have passed since the bacteria were shed. This suggests that 99% of the bacteria will be dead after 385 days, in concordance with Whittington et al. (20).

#### Contamination between Farm Sections

We also simulate the spread of MAP between farm sections on the dirty boots of personnel and wheels on machines (including contaminated tools). For each time step, the amount of cross-contamination of MAP from each section is calculated as:

(5) $${\mathrm{Spillover}}_{\text{j}}=\left(\exp\left(\frac{-1}{N_{\text{P}}}\right)\cdot S_{\text{P}}+\exp\left(\frac{-1}{N_{\text{M}}}\right)\cdot S_{\text{M}}\right)\cdot {\mathrm{MAP}}_{\text{j}}$$

where *N*_P_ and *N*_M_ are the numbers of personnel and machines, respectively, that can potentially transmit MAP between farm sections, *S*_P_ and *S*_M_ is the level of cross-contamination from boots and machines, respectively, and *MAP*_j_ is the amount of MAP shed in farm section j. Cross-contamination of MAP from all four sections is summed daily and divided equally between the sections to simulate an even spread of MAP on the farm. A machine on the farm by default takes 3% of the bacteria shed from all farm sections (maximum 8% of shed MAP can stick to machines) and divides it evenly into all sections again. The default for one farm worker is 0.3% of daily shed bacteria in each section (maximum 1% of shed MAP can stick to personnel).

#### Risk of Infection from the Environment

The daily risk of obtaining an infection for the individual animals is modeled through the environment:

(6) $$R_{\text{j}}=1-\left(1/\exp\left(\left(1/H\right)\cdot F\cdot {\mathrm{MAP}}_{\text{j}}\right)\right)$$

where *R*_j_ is the probability of each animal of acquiring an infection with MAP shed in farm section j, *H* is the hygiene level on the farm, *F* is the force of infection parameter, which was calibrated in the model (see below). *MAP*_j_ is the amount of MAP shed in section j. MAP can thus accumulate in each farm section, but the survival of the bacteria decreases with time. It is possible to adjust the force of infection in the model, thus increasing or decreasing the risk of infection from the environment.

The hygiene level represents the likelihood that MAP will find a surface to stick to in the farm section, i.e., in principle a proxy of how clean the stable is.

#### Risk from Other Transmission Routes

Within the model, there are different transmission routes: *in utero* infection, infection from MAP in the environment of each farm section, infection from colostrum, and infection from waste milk. For *in utero* infection, we used the estimates from Whittington and Windsor (21), but for the latter three, we did not have a direct estimate from the literature. However, a previous Danish study (22) estimated that the annual reduction in the odds ratio of infection when calves were not fed waste milk from repeatedly test-positive cows was −0.05. Over a 5-year period, this effect corresponds to an odds ratio of exp(−0.05⋅5) = 0.78 (CI: 0.65–0.95). Similarly, the effect of not using colostrum from repeatedly test-positive cows was exp(−0.04⋅5) = 0.82 (CI: 0.67–1.00). In this model, we set the risk of infection from waste milk to 0 when the calves are not fed with waste milk from repeatedly test-positive cows. Likewise, we set the risk of infection from colostrum to 0 if the farmer did not feed calves with colostrum from repeatedly test-positive cows. To adjust waste milk risk, colostrum risk and force of infection, we ran a series of simulations [with 500 iterations, 3-year burn-in period (initial simulation time required to stabilize the system) + 5 simulation years and 5.6% initial prevalence], varying these levels (data not shown). This gave a 3D parameter space for calibrating the model. We then chose the set of parameters that came closest (based on visual inspection) to (1) keep the true prevalence stable at about 5.6% over the five simulated years, (2) yield a 22% lower apparent prevalence when not feeding calves with waste milk from repeatedly test-positive cows, and (3) yield a 18% lower apparent prevalence when not using colostrum from repeatedly test-positive cows. This approximation was deemed appropriate for calibrating the three levels of infection in the model to maintain a stable endemic status of MAP in the farm. If the farmer removed the calves from the dam at birth, the risk of infection from the dam was reduced by 95%, causing the apparent prevalence to drop to about 0.80% compared with no calves being removed. This was within the confidence limits for the estimated effect of this control action which was previously estimated to reduce the risk of infection to 0.70% (0.53–0.95% CI) of the previous level (22). We did not want to reduce this risk to 0 when the control action was implemented, since the calf still faces some risk of infection from the dam *via in utero* transmission.

#### Model Calibration

**Figure 1:**
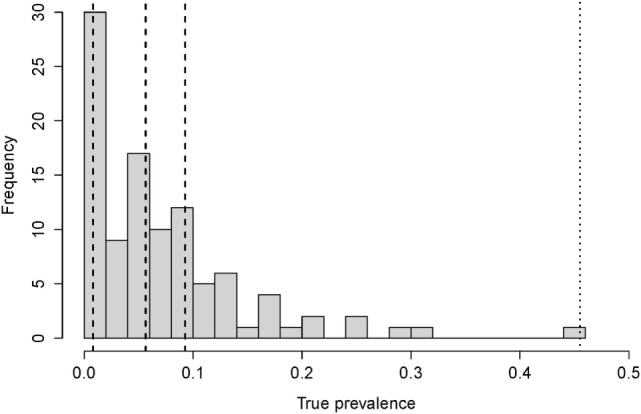
**The distribution of the true prevalence calculated for 102 randomly chosen herds in Denmark**. The dashed lines show the 25, 50, and 75 percentiles (at 0.8, 5.6, and 9.3% prevalence, respectively), and the dotted line show the maximum observed prevalence (45%).

In the model, we record both true and apparent prevalence within the herd. The true prevalence is observed from the number of infected (adult) cows, in all states of disease. The apparent prevalence is calculated from the number of (adult) cows that are test-positive. We used the maximum of the estimated prevalence (45% prevalence) to determine a low-hygiene scenario in a herd with high prevalence, hereafter referred to as the low-hygiene herd (Figure 1).

We calibrated the force of infection in the model, so the prevalence was stable over five simulated years. This time span was chosen because the estimated effects (odds ratios) of the control actions were based on a maximum of 4 years (22). Paratuberculosis has been present in Denmark for many years. Therefore, we assume that the within-herd prevalences have stabilized within each farm. Furthermore, we found no evidence in our data (Figure 1) of within-herd prevalences >45%. Even if some farms have such a high prevalence, they would be rare, and our aim was to represent average Danish farms.

We based our “no control” (baseline) scenario on the dataset of 102 farms described above. Age-specific sensitivity estimates along with the specificity (23) were used to estimate the true prevalence within these herds, using the approach described in Sergeant et al. (24). The resulting prevalences are shown in Figure 1.

#### Calves

At calving, calves have a probability, *P*_j_, of becoming infected:

(7) $$P_{\text{j}}=1-\left(1-D\right)\left(1-S\right)\left(1-\left(R_{\text{j}}\left(1-C\right)\right)\right)$$

where j denotes the (calving) section, *D* is the probability of infection from the dam to the calf: 9% for calves born from subclinical cows (states: low-shedding and high-shedding), and 39% from clinical cows (state: affected or clinical) [(20); Table S1 in Supplementary Material]. *S* is the reduction in the risk of infection from the dam to the calf when they are separated, corresponding to 0 if calves are not removed from the dam within 2 h from birth. *R* is the risk of obtaining an infection from the environment. *C* is the fractional reduction in the MAP shed within the calving area if the farmer cleans between calvings, default set to 1. See below, for the calibration of these parameters.

If the calf is removed from the dam within 2 h after calving, *D* is decreased by 7% [default; taken as the difference in risk between not removing any calves, and removing calves from cows identified as infected (22)]. We used 4% (default) risk of this if the individual calving pens were not cleaned between calvings [taken as the difference in risk between not cleaning calving pens and cleaning calving pens with repeatedly test-positive cows (22)]. The susceptibility of newborn calves to infection is equal to 1. A risk calf (i.e., a calf born to a dam with antibodies) is back-traced if their dam delivers a positive ELISA within 200 days after calving, enabling a strategy where risk calves can be culled (25).

After calving, the calves have a daily probability of becoming infected:

(8) $$P_{\text{j}}=\left(1-\left(1-R_{\text{j}}\right)\left(1-R_{\text{M}}\cdot F\right)\right)\cdot \mathrm{Susc}\left(\mathrm{age}\right)$$

where *P*_j_ is the cumulated probability of infection from MAP in section j, here the calf section, *R*_j_ is the risk of infection from MAP shed in farm section j, *R*_M_ is the risk of infection from colostrum when the calf is 1–3 days old or from waste milk when the calf is between 3 days and 8 weeks old. *F* is the fraction of lactating cows that are infected at that time (and therefore able to infect *via* colostrum or waste milk), and *Susc*(*age*) is the age-dependent susceptibility, equal to 1 for newborn calves.

#### Heifers and Cows

Heifers, lactating cows, and dry cows have a daily probability, *P*_j_, of becoming infected:

(9) $$P_{\text{j}}=R_{\text{j}}\cdot \mathrm{Susc}\left(\mathrm{age}\right)$$

where *R*_j_ is the risk of infection from MAP shed in section j, where j can be any one of the four sections: heifers, lactating cows, dry cows, or the calving pen. *Susc*(*age*) is the susceptibility depending on the age of each animal.

Traditionally, calves have been perceived as most susceptible to MAP, but recent research has shown that animals older than 1 year are also susceptible to MAP infection (26, 27). In this model, we constructed the susceptibility of each animal to MAP given by this function:

(10) Susc(age)=exp(−0.01⋅age)

where 0.01 is a scaling coefficient, and *age* is measured in years. In this way, the susceptibility to infection drops exponentially to 2.6% at the age of 1 year and 0.07% at the age of 2 years. Thus, there is a small risk of infection for older animals.

### ELISA

We incorporated cow-specific results based on the ELISA in the model. Milk ELISA is done quarterly in all herds participating in the Danish MAP control program. The sensitivity of the ELISA is based on Nielsen et al. (23) and is a logarithmic function dependent on the age of the tested animal, resulting in an ELISA value indicating if the animal is infected.

To simulate the test strategy currently used in Denmark, cattle in the different states of MAP infection are tested every 3 months. Animals that are susceptible to MAP are assigned a test value for the ELISA reading taken from a uniform distribution between 0 and 0.30405 (to simulate a specificity of 98.67%). The cut-off for identifying a cow with antibodies is ≥0.30, and test values above 0.30 for susceptible animals are considered technical variation. The test value for an animal in state 1 (infected and low-shedding) and state 2 (infected and high-shedding) is given by:

(11) $$\mathrm{TV}\left(\mathrm{age}\right)=U\left[\mathrm{Cut}-\left(\left(1-\mathrm{Sens}\left(\mathrm{age}\right)\right)\cdot 4.5\right);\;\mathrm{Cut}\right.\left.+\left(\mathrm{Sens}\left(\mathrm{age}\right)\cdot 4.5\right)\right]$$

where *TV* (*age*) is the test value dependent on the age of an animal, *U* is a uniform distribution defined by [min; max], *Cut* is the cut-off used to determine positive tests, and *Sens*(*age*) is the sensitivity for the ELISA dependent on age of the tested animal, based on Nielsen et al. (23). The min and max of the uniform distribution are calculated, so that the proportion of the interval above the cut-off value is the same as the age-sensitivity for a given animal. In this way, the ELISA value is adjusted to the age of each tested animal and to the specific cut-off value. For example, animals at the age of 1, 2, 3, 4, and 5 years have the probabilities of 3, 27, 54, 68, and 74%, respectively, of a positive result. If the uniform distribution yields a negative test value, it is converted into 0 in concordance with real ELISA values. The test value 4.5 is introduced to create a maximum value of 4.5 test value units above the cut-off, reflecting real ELISA values.

Animals in the affected stage of infection get a test value taken from a uniform distribution between 0.5 and 5 and will therefore always be positive. Every 3 months, the animals are separated into antibody groups based on the repeated recordings of the last four test results, as described by Nielsen (25).

### Economics

Infected animals are subjected to a reduction in slaughter value due to weight loss if they have tested positive in at least one of the last three tests (28). Therefore, cows with fluctuating responses lose 12.9% of their slaughter value, those with only the latest test-positive lose 7.9% of the slaughter value, and those with repeated positive ELISAs lose 16.6% of their slaughter value. The relative ECM yield level in infected cows is reduced according to the latest ELISA value (29), where we describe the daily milk reduction, *MR*, in ECM by:

(12) $$\mathrm{MR}=\left\{\begin{array}{ccc} 2/3{\mathrm{TV}}^{2}-2/5\mathrm{TV}+1.02 & , & \mathrm{TV}<0.3\\ 0.96 & , & 0.3\leq \mathrm{TV}<0.9\\ 1-0.044\mathrm{TV} & , & 0.9\leq \mathrm{TV} \end{array}\right.$$

Milk production and the income from slaughtered cows are summarized both as measures corrected for ELISA values and uncorrected measures for comparison with a MAP-free scenario. For each simulation, the number of ELISAs performed, bull calves sold, carcasses destroyed, and inseminations conducted are summarized, as are the man-hours spent cleaning calving boxes (1 h per calving), handling colostrum (2 h per test-positive cow calving), and handling calves if removed immediately following birth (1 h per test-positive cow calving). The model also summarizes the daily amount of money spent on feed (see Feeding). The prices of milk and labor costs per hour are listed in Table S1 in Supplementary Material.

For each simulated scenario, we calculated the change in net revenue annually by subtracting the expenses (feed, labor, inseminations, and destructions) from the income (milk production, sold bull calves, and slaughtered cows). To obtain the yearly change in net revenue per cow year, we also divided the net revenue by the annual number of cow years for a comparison of the simulated scenarios. We chose to use cow years for comparability with other studies even though the number of cows in the simulated herd varies only slightly over the years. For all scenarios, we also report total net revenue (the sum of net revenue over 10 years). We did not consider the development of value over time; that is, we assume a 0% discount rate.

### Test Herd Generation

We generated a test herd to examine the model performance and evaluate the test scenarios. The test herd represents a medium-size Danish dairy herd with 118 calves (age 0–1 year), 127 heifers (age 1–2.5 years), and 200 cows (age 2–7 years) (Table S3 in Supplementary Material). The milk level for each cow was randomly assigned from a distribution estimated based on the dataset. A number of animals in the test herd were initially infected from the beginning of the simulations according to the specified prevalence. The number of initially infected animals and their progression through disease states were randomly chosen for every simulation. The number of days spent in the assigned disease state was drawn from a normal distribution with mean equal to the corresponding value taken from expert opinion (Table S2 in Supplementary Material).

For comparison of the results, we set the seed to a new value at the beginning of each iteration. We used the same string of seeds on all test scenarios to allow comparisons.

### Impact of MAP Control Actions

We used the model to examine the epidemiological and economic impact of four of the seven recommended actions to control and prevent infection with MAP in dairy cattle herds (22, 25). The actions are built upon a classification system where cows are divided into “red,” “amber,” and “green” groups. “Red” cows have tested positive a minimum of two times within the last four tests (repeatedly test-positive cows), “amber” cows have tested positive at least once in the last four tests, and “green” cows have only tested negative in the four most recent tests (25).

The four evaluated actions are described below, including implementations, costs, and impact:

(1) Remove calves from “red” and “amber” cows within 2 h of calving. Of 1081 farmers in the control program, 736 (68%) stated that they had implemented this practice (22). It decreased the apparent within-herd prevalence to 70% of the initial prevalence over a 5-year period (22) and is estimated to cost one man hour per calving.
(2) Avoid feeding colostrum from “amber” or “red” cows to calves or pasteurize colostrum. Of 1081 farmers in the control program, 707 (65%) claimed to do this (22). It was estimated to decrease the apparent within-herd prevalence to 82% of the initial prevalence over a 5-year period (22). The financial cost of this action has been set to 0.
(3) Avoid using waste milk from “amber” or “red” cows for feeding calves. Of 1081 farmers, 742 (69%) in the Danish control program implemented this, causing the apparent prevalence to decrease to 78% of the initial prevalence over a 5-year period (22). This action has been set to have no direct financial cost.
(4) If “red” cows are not allowed to calve, they get a flag on the culling list and are therefore prioritized for voluntary culling. This action has no direct economic cost and the impact is a direct output of the model.

### Herd Hygiene: Average vs. Low

For each control scenario, we used a generalized test farm that resembled the average Danish farm with 200 cows. In all simulations, we used a burn-in period of 3 years to stabilize the herd (especially with regard to build-up of MAP in the environment) before any actions are implemented. We initiated all scenarios with 5.6% prevalence and repeated the simulations 500 times which was found to be adequate in the convergence test (Figure S1 in Supplementary Material).

For the average-hygiene herd, we set the hygiene level to 1 in this scenario, stabilizing the true prevalence at a median of 6% within the simulated herd in the baseline scenario. For all other MAP-related parameters, default values were used (Table S2 in Supplementary Material). In this study, we simulated the following scenarios:

(1) No actions against MAP infection are implemented (baseline scenario).
(2) The three most popular actions (1–3 as described above), i.e., those that were implemented by more than 50% of the participating farmers in the control program (22, 25) are implemented. We make the assumption that farmers implementing an action for all possibly infectious (“amber”) cows will also implement it for cows identified as likely to be infectious (“red” cows).
(3) The farmer implements actions 1–3 (as described above) in addition to culling the cows identified as infectious (“red” cows).
(4) The farmer only implements action 1.
(5) The farmer only implements action 2.
(6) The farmer only implements action 3.
(7) The farmer places only implements action 4.

We also simulated a herd with a prevalence of 45% (the highest herd prevalence found in the 102 herds with no control) for comparison with the average-hygiene herd. The initial prevalence was set to 45%, and the hygiene level was adjusted to 0.806. In a sensitivity analysis, this hygiene level was able to sustain a median prevalence of 45% over 10 years (data not shown). This low-hygiene herd reflects a scenario where the hygiene level, MAP build-up and general properties of the farm and management cause the prevalence to persist at 45%. As in the average-hygiene herd, we allowed a 3-year burn-in period for MAP to build up in the farm sections. We also used the same number of animals and the same age demographics as assumed for the average-hygiene herd.

### Model Validation

#### Internal Validation

We internally validated the model using the rationalism method (checking the consistency of results and comparing results with different inputs), the tracing method (following single animals and their properties over time), unit testing (where cow attributes were observed and controlled during model iteration), and the face validity method (where the code was revised for functionality and all input parameters scrutinized) (30).

#### External Validation

We compared the true prevalence predicted by the model to the true prevalence from the dataset of 102 farms without control measures. In this way, we validated the baseline scenario using field data.

#### Convergence Test

In order to determine the required number of iterations, we conducted a convergence test on the median net revenue estimate from the model. We deemed that 500 iterations were sufficient to reach a stable variance of the estimates as determined by visual inspection (see Figure S1 in Supplementary Material).

#### Sensitivity Analysis

We tested 38 parameters in sensitivity analyses to assess the robustness of the model with regard to the prevalence, milk yield, and economic output. The parameter names are described in detail in Tables S1, S2, and S4 in Supplementary Material.

## Results

The results of the simulations for an average-hygiene herd and a low-hygiene herd are summarized in Tables 1 and 2. Extensive results about the epidemiological production and economic results of the seven scenarios, and the sensitivity analysis are shown in Supplementary Materials. In this section, we cite median results unless otherwise stated. In the figures, we show the 50% simulation envelope for the results, corresponding to the outcome between the 25th and 75th percentiles.

**Table 1:** **Results of the scenarios on an average-hygiene herd with a baseline true within-herd prevalence of 5.6%**.

Scenario			
	ECM (5%; 95%)	TP (5%; 95%)	AP (5%; 95%)
No control	20.12 (19.90; 20.39)	7.02 (0.00; 18.46)	5.91 (1.46; 13.81)
Three actions scenario	20.15 (19.88; 20.40)	2.43 (0.00; 8.29)	2.93 (0.49; 7.32)
Four actions scenario	20.17 (19.90; 20.42)	0.00 (0.00; 0.00)	0.99 (0.00; 2.46)
Remove calves	20.13 (19.88; 20.37)	5.91 (0.00; 15.20)	5.37 (1.42; 12.25)
Handle colostrum	20.14 (19.88; 20.41)	5.33 (0.00; 16.02)	4.87 (0.98; 12.68)
Handle waste milk	20.13 (19.89; 20.39)	4.87 (0.00; 12.75)	4.41 (0.99; 10.85)
Cull pos. cows	20.16 (19.91; 20.41)	0.00 (0.00; 0.00)	1.44 (0.00; 2.46)

	**EXP (5%; 95%)**	**INC (5%; 95%)**	**TNR (5%; 95%)**

No control	5.10 (5.05; 5.16)	8.12 (7.99; 8.25)	3.02 (2.91; 3.12)
Three actions scenario	5.21 (5.14; 5.27)	8.13 (8.00; 8.26)	2.92 (2.82; 3.02)
Four actions scenario	5.20 (5.14; 5.26)	8.14 (8.01; 8.27)	2.94 (2.84; 3.03)
Remove calves	5.20 (5.14; 5.26)	8.12 (7.99; 8.25)	2.93 (2.83; 3.02)
Handle colostrum	5.14 (5.07; 5.20)	8.13 (7.99; 8.26)	2.99 (2.88; 3.08)
Handle waste milk	5.13 (5.07; 5.20)	8.13 (7.99; 8.26)	2.99 (2.88; 3.09)
Cull pos. cows	5.13 (5.07; 5.20)	8.15 (8.01; 8.26)	3.01 (2.91; 3.09)

*ECM, kilograms of ECM milk yield from of all cows on the farm; TP, true within-herd prevalence; AP, apparent within-herd prevalence; EXP, expenses in million €; INC, income in million €; TNR, total net revenue in million € over 10 years*.

*The numbers are median results with 5 and 95% confidence limits, calculated over the 10 simulated years. Milk yield and economic values are shown in millions. Prevalences shown in % are the resulting prevalences at the end of the simulations*.

**Table 2:** **Results of the scenarios on a low-hygiene herd with a baseline true within-herd prevalence of 45%**.

Scenario			
	ECM (5%; 95%)	TP (5%; 95%)	AP (5%; 95%)
No control	19.83 (19.57; 20.08)	38.73 (28.43; 47.80)	27.32 (19.60; 34.32)
Three actions	19.90 (19.66; 20.16)	19.61 (10.78; 28.30)	15.12 (8.33; 22.06)
All actions	20.03 (19.76; 20.32)	0.00 (0.00; 0.00)	0.99 (0.00; 2.48)
Remove calves	19.84 (19.59; 20.08)	34.31 (22.80; 44.35)	24.15 (16.33; 31.53)
Handle colostrum	19.84 (19.60; 20.08)	33.25 (22.69; 43.00)	23.96 (15.76; 31.26)
Handle waste milk	19.86 (19.61; 20.12)	30.64 (19.99; 41.48)	22.44 (14.29; 30.40)
Cull pos. cows	20.01 (19.75; 20.29)	0.00 (0.00; 1.47)	1.46 (0.00; 2.96)

	**EXP (5%; 95%)**	**INC (5%; 95%)**	**TNR (5%; 95%)**

No control	5.04 (4.97; 5.10)	8.00 (7.86; 8.13)	2.97 (2.86; 3.05)
Three actions	5.17 (5.10; 5.23)	8.03 (7.90; 8.16)	2.87 (2.76; 2.95)
All actions	5.16 (5.09; 5.23)	8.11 (7.97; 8.23)	2.95 (2.85; 3.03)
Remove calves	5.14 (5.07; 5.20)	8.01 (7.87; 8.12)	2.87 (2.77; 2.95)
Handle colostrum	5.09 (5.04; 5.15)	8.01 (7.87; 8.14)	2.92 (2.81; 3.01)
Handle waste milk	5.07 (5.01; 5.14)	8.02 (7.88; 8.14)	2.94 (2.85; 3.03)
Cull pos. cows	5.08 (5.01; 5.15)	8.10 (7.97; 8.23)	3.02 (2.93; 3.11)

*ECM, kilograms of ECM milk yield from of all cows on the farm; TP, true within-herd prevalence; AP, apparent within-herd prevalence; EXP, expenses in €; INC, income in €; TNR, total net revenue in million € over 10 years*.

*The numbers are median results with 5 and 95% confidence limits, calculated over the 10 simulated years. Milk yield and economic values are shown in millions. Prevalences shown in % are the resulting prevalences at the end of the simulations*.

### Average-Hygiene Herd

**Figure 2:**
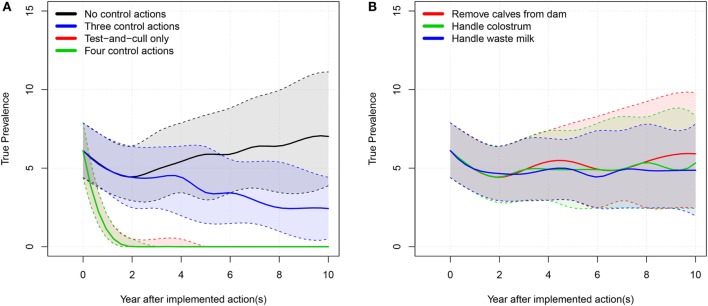
**True prevalence: 50% simulation envelope over 10 simulated years for the tested scenarios in the average-hygiene herd**. **(A)** “Three control actions” means the three control actions in **(B)**. “Four control actions” means the three actions in B plus test-and-cull. **(B)** “Handle waste milk” and “handle colostrum” means that the farmer only uses milk or colostrum from test-negative cows for feeding calves.

We show the results of the average-hygiene herd simulations in Table 1. Milk yield, income, and expenses are cumulated over the simulated 10-year period. The true prevalence and apparent prevalence shown are the end prevalences after 10 simulated years. The true prevalence is shown over time for the average-hygiene herd in Figure 2.

When all four examined actions against MAP were implemented, the model showed that it was possible to eradicate MAP from the farm. When only the test-and-cull strategy was implemented, it was also possible to eradicate MAP (i.e., to reduce true prevalence to 0). When the three most popular actions were implemented, true prevalence was reduced to a median level of 2.4%. There was only a marginal reduction in prevalence when the actions of removing calves, handling colostrum, and handling waste milk were implemented independently.

The best scenario judged using the mean milk production was the one where all actions were implemented, yielding a total of 20.17 million kilograms of ECM over the 10 simulated years (Table 1). The lowest milk production was observed when no control actions were implemented, yielding 20.12 million kilograms of ECM.

**Figure 3:**
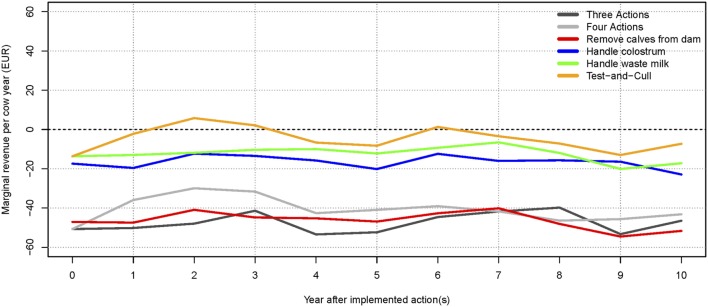
**Change in net revenue per cow year over time for the average-hygiene herd, relative to the baseline scenario**. The marginal for each action and combinations are shown. The dotted line (at 0€) represents the baseline scenario (no control).

The scenario where no control actions were implemented generated the highest total net revenue (summed over 10 years), followed by the test-and-cull strategy. The lowest total net revenue was found when the three most popular actions were implemented. This is a result of the higher expenses for implementing these actions, which are not offset by sufficiently higher revenues (results not shown). The scenario with the highest (and intermittently positive) net revenue per cow year was when only a test-and-cull strategy was implemented (Figure 3). All other scenarios consistently yielded negative change in net revenue per cow year during the 10 simulated years.

The apparent prevalence was slightly lower than the true prevalence in most of the scenarios for the average-hygiene herd (Table 1). However, when the prevalence was very low, for instance, when three actions were implemented, the apparent prevalence was higher (2.93) than the true prevalence (2.43). This is caused by the specificity of the test resulting in false positive results.

### Low-Hygiene Herd

**Figure 4:**
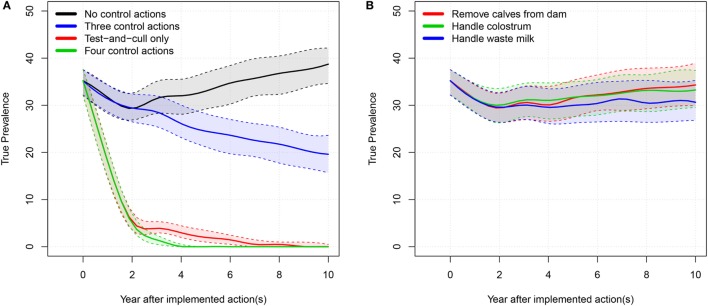
**True prevalence: 50% simulation envelope over 10 simulated years for the tested scenarios in the low-hygiene herd**. **(A)** “Three control actions” means the three control actions in **(B)**. “Four control actions” means the three actions in **(B)** plus test-and-cull. **(B)** “Handle waste milk” and “handle colostrum” means that the farmer only uses milk or colostrum from test-negative cows for feeding calves.

The results of the simulated low-hygiene herd are shown in Table 2. Milk yield, income, and expenses are cumulated over the simulated 10-year period. The true prevalence and apparent prevalence shown are the end prevalences after 10 simulated years, while the development is illustrated in Figure 4. The model was calibrated over 5 years to fit a median prevalence level of 45% in a scenario where no control actions were implemented. The prevalence then decreased to 39% over 10 years. This effect was seen because we calibrated the model to be stable over 5 years but used it to predict for 10 years and therefore the prevalence will change over longer periods (Table 2).

In the low-hygiene scenario, as was true for the average-hygiene herd, it was only possible to eradicate MAP from the herd by using all four actions or by using a test-and-cull strategy alone. The three most popular actions did not have considerable impact when implemented independently, but reduced the median true prevalence to 20% when combined.

Again, when considering milk production, the best scenario was the one where all actions were implemented, followed by the test-and-cull strategy (Table 2). The lowest milk production was reached when no control actions were implemented.

The number of cow years was kept stable throughout all simulations, with a mean of 205 cow years (min: 203, max: 208). Here, we report the revenue per cow year to ease comparison with other management actions and herd sizes.

**Figure 5:**
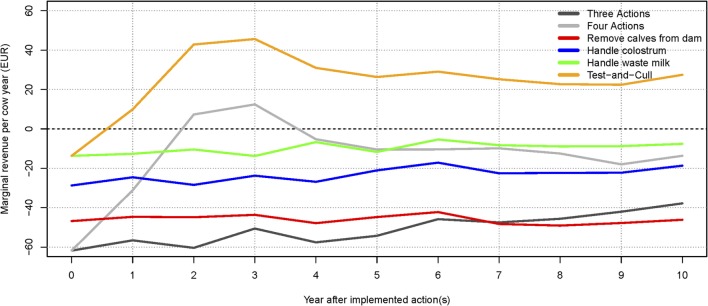
**Change in net revenue per cow year over time for the low-hygiene herd, relative to the baseline scenario**. The marginal extra income for each action and combinations are shown. The net revenue of implementing the three most popular actions (handling colostrum and waste milk, and removing calves from the dam) increases after 6 years but is still not profitable. The dotted line (at 0€) represents the baseline scenario (no control).

The highest total net revenue (summed over 10 years), at 3.02 million €, was attained in the scenario where test-positive cows were culled. This was largely due to an increased income from a higher milk yield and the higher slaughter value of healthy cows. The highest income came from the scenario where all actions were implemented (8.11 million €), but this was counterbalanced by an increase in the expenses for the actions (5.16 million €). The lowest expenses were in the scenario where no control or handling of waste milk was implemented, but these were counterbalanced by lower incomes. The scenario generating the second-highest net revenue on average was when no control actions were implemented. Scenarios generating the lowest net revenues were when three actions were implemented, or when calves were removed from potentially infectious dams. It was, therefore, more profitable on average to avoid implementing control actions, rather than implement the three most popular actions on their own. The test-and-cull scenario consistently yielded a positive change in net revenue per cow year over the 10 simulated years (Figure 5). The scenario with all four actions implemented had positive change in net revenue per cow year in years 2 and 3 but was otherwise negative. The scenario with three actions showed steadily increasing net revenue per cow year after 4 years, yet it was still negative after 10 simulated years. All other scenarios had negative net revenue per cow year.

### Sensitivity Analyses

The extensive results of the sensitivity analyses are shown in Tables S5–S10 in Supplementary Material. The parameters that had a negative correlation with the true prevalence were heat detection success for heifers; insemination success for heifers and cows; cross-contamination from boots and machines; duration of the low-shedding and high-shedding infection stages; and hygiene level. The negative impact of higher cross-contamination is likely due to a higher proportion of the shed MAP being spread out on the farm, thus lowering the local probability of infection in each farm section. Parameters with a positive correlation to the true prevalence were the maximum number of heat cycles before culling, the percentage of voluntary culling, the amount of bacteria shed in all infection stages, the proportion of stillbirths, and the duration of the affected stage. The positive impact of more voluntary culling on the true prevalence is likely caused by a change in the demography of the herd, leading to higher transmission (no test-and-cull in this scenario). The test specificity did not seem to impact the true prevalence (no test-and-cull in this scenario), but was negatively correlated with the apparent prevalence. Lowering the level of the test sensitivity to 50% so that the sensitivity for 5-year-old cows was 37% resulted in a slightly increased true prevalence (from 7.02 to 7.28 median result) prevalence after 10 simulated years. Increasing the test sensitivity level to 120% so that it was 88% for 5-year-old cows was able to decrease the true prevalence from 7.02 to 6.83% (median result).

## Discussion

Our results showed that on average the most economically profitable strategy for a low-hygiene herd was to cull “red” and “amber” cows (Figure 5), resulting in eradication of the disease within 7–10 years (50% simulation envelope, Figure 4). However, it was not possible to eradicate MAP in all of the simulations within 10 years by using the test-and-cull strategy (Figure 4), where the true prevalence was still 1.47% at the 95% percentile (Table 2). For this scenario, 69% of the simulations resulted in zero prevalence after 10 years (data not shown). Therefore, we conclude that, although test-and-cull is the most profitable control action for low-hygiene herds, it is not guaranteed to eradicate MAP. Future studies should investigate whether eradication could be guaranteed by combining test-and-cull with some preventive measure (other than those already analyzed here), and whether this approach would be more profitable than test-and-cull alone.

In herds with average hygiene, test-and-cull is sufficient for eradication, but not more profitable than no control. Therefore, we suggest that in these herds, test-and-cull is used if the aim is to eradicate paratuberculosis or lower the prevalence. Because the effect of the simulated control measures in herds with average hygiene is limited, and because the costs are considerable, we suggest that these herds focus on test-and-cull alone.

We found that implementing only one of the three most popular control actions did not have much impact on the prevalence (Figures 2B and 4B). However, there was a synergistic effect of implementing all three actions at the same time (Figures 2A and 4A). Therefore, it is not economically attractive to implement just one of these actions due to the associated cost, which is not counterbalanced by enough benefit. And although a combination of these three control actions reduces prevalence more effectively, such a combination is among the least profitable strategies (Figures 3 and 5). This is concordant with the results from the SimHerd model (6).

When considering only the prevalence of MAP, we found that the optimal scenario was to implement all control actions, allowing the farmer to eradicate MAP from the farm completely in the average-hygiene herd within 3 or 5 years (50% simulation envelope, Figures 2 and 4, respectively). This assumes that MAP is not reintroduced into the farm at any point. This scenario was also the most expensive (Table 2).

The results of this study contradict results of previous models that have presented within-herd true prevalences between 50 and 90% if no control actions are implemented on the farm (6, 8–11). We did not find such high within-herd prevalences in Denmark, but found a median within-herd true prevalence at 5.6% and a maximum within-herd true prevalence of 45% (Figure 1). This is close to the result of Verdugo et al. (4) who estimated the median within-herd true prevalence in Danish farms to be 7%. The reason for the difference between our results and those of previous models is mainly because we calibrated the model to keep a stable prevalence. This constraints the transmission process in the model, so the prevalence is not able to increase exponentially.

In both the low- and average-hygiene herd, the best action to reduce the prevalence was to cull test-positive cows, supporting the findings of Nielsen and Toft (22). However, this contradicts the findings from JohneSSim and SimHerd simulations, where it was found that test-and-cull strategies could not lower the prevalence and that it was not economically attractive (6, 31). However, in the SimHerd model, an ELISA-positive cow must be confirmed by a fecal culture. This is a more time consuming and expensive test than the ELISA used in the PTB-iCull model, where positive cows can be put on top of the culling list as soon as they are detected. The JohneSSim model simulated a low ELISA sensitivity based on the disease state of the animal contrary to the disease state and age-dependent sensitivity used in the current model. This could add to the differences between the results of our and previous models.

Previous models of MAP spread showed that it is impossible to eradicate MAP even with the use of rigorous test-and-cull strategy [e.g., Ref. (32)], which contradicts our results. The way we model MAP spread is different than in earlier work [see review by Marcé et al. (33)]. In those models, the probability of infection through the environment is a function of, among others, the number of infectious animals in the herd (frequency models) in a Reed-Frost model (33). In our work, we use a density-dependent transmission model to estimate the probability of infection through the environment, depending on density of the bacterial load in the environment. Density-dependent models tend to represent endemic situations better than frequency-dependent models that tend to seek pathogen/host extinction (12). In frequency-dependent models, when no control actions are taken, the prevalence often increases sharply to reach unusually high levels, as predicted by previous models. Furthermore, in frequency-dependent models, infectious cows that are culled immediately cease to be infective, whereas in our model, MAP is shed in the environment and can still give new infections if the shedding cow was culled earlier. As discussed above, we observed a median within-herd true prevalence of 5.6% in herds that have no control actions against MAP and have been practicing for several years (data not shown), indicating a stable endemic state of MAP in these herds. We, therefore, consider a density-dependent model more representative of actual field situation than a frequency-dependent model that would lead to massive spread of MAP.

In addition, our model differs from previous models in that the sensitivity of the ELISA is higher than those used in previous models, as we use more recent estimations based on Nielsen et al. (23).

An important difference between our and previous models is also that we model a closed herd with no risk of disease introduction through animal purchase. The reason we model a closed herd is that about 50% of the herds in Denmark are closed (data not shown), and that it is recommended to keep the herd closed to avoid introduction of MAP and other diseases. Simulating an open herd would prevent eradication because of the risk of continuous introduction of infected animals.

The relationship between susceptibility and age has not yet been fully established, so we chose this function to incorporate a small probability of infection even for old animals, as described earlier (26). Susceptibility to MAP is also influenced by genetic variation (34), but incorporating this into the simulation model would require simulating genetic profiles of each cow, which is out of the scope of the current study.

The findings of this study may be representative for other countries than Denmark. However, care must be taken, when translating the results to other countries, because certain key parameters, such as prevalence, interest rate, and control options, might differ between countries.

Further research should focus on investigating the relationship between bacterial load and force of infection, as this relationship might not be linear, as suggested by Slater et al. (35).

## Conclusion

We used current knowledge of MAP infection and detection mechanisms to build a new framework for simulating MAP infection within a herd. We simulated the epidemiological and economic effects of different control strategies in a average and a low-hygiene herd. The most profitable scenario over 10 years in the average-hygiene herd was to avoid implementing a control strategy. In the low-hygiene herd, a test-and-cull strategy was the best solution economically. We did not find it profitable to implement any of the three most popular actions for preventing the spread of MAP within herds in Denmark, either for the low or the average-hygiene herds. The results will help farmers improve control of MAP in their herds.

## Author Contributions

CK, KG, TH, SN, LC, and NT developed the model. ER provided knowledge and data and participated in the model formulation. CK wrote the first draft of the manuscript.

## Conflict of Interest Statement

The authors declare that the research was conducted in the absence of any commercial or financial relationships that could be construed as a potential conflict of interest.
