# Supplementary material for: Simulating the Epidemiological and Economic Impact of Paratuberculosis Control Actions in Dairy Cattle
Source: Front Vet Sci. 2016 Oct 10;3:90. doi: 10.3389/fvets.2016.00090 (PMC5056316; doi:10.3389/fvets.2016.00090)
Supplement: Supplementary file 1 [file Data_Sheet_1.docx]

**Supplementary Materials**

**Table 3.** Probabilities, durations of periods and prices used in the model. Durations (except for calfhood) are described by *N*(*x, sd*), a normal distribution around the mean *x* with the standard deviation *sd*. All prices are in EUR

| Parameter | Default value | Explanation | Reference |
| --- | --- | --- | --- |
| Heat Detection (Cow) | 0.36 | Probability of detecting heat (cows) (per heat period) | (Ancker et al., 2009) |
| Pregnancy Probability (Cow) | 0.42 | Probability of pregnancy (cows) (per insemination) | (Ancker et al., 2009) |
| Heat Detection (Heifer) | 0.60 | Probability of detecting heat (heifers) (per heat period) | (Ancker et al., 2009) |
| Pregnancy Probability (Heifer) | 0.55 | Probability of pregnancy (heifers) (per insemination) | (Ancker et al., 2009) |
| Days Calf | 365 | Number of days spent as a calf | Expert opinion |
| Days Heifer | *N* (110*,* 2) | Number of days spent as a heifer | Expert opinion |
| Days Inseminated Heifer | *N* (41*,* 2) | Number of days to spend as inseminated heifer | Expert opinion: Pregnancy can be tested after 41 days. |
| Days Pregnancy Heifer | *N* (280*,* 2) | Number of days to spend as pregnant heifer | Expert opinion |
| Days Milk Cow | *N* (21*,* 2) | Number of days to spend as a lactating cow between inseminations | Expert opinion |
| Days Post Calving | *N* (40*,* 2) | Number of days spent as a cow between calving and insemination | Expert opinion |
| Days Inseminated Cow | *N* (41*,* 2) | Number of days spent as inseminated cow | Expert opinion: Pregnancy can be tested after 41 days. |
| Days Pregnancy Cow | *N* (224*,* 2) | Number of days spent as pregnant cow | Expert opinion |
| Days Dry Cow | *N* (56*,* 2) | Number of days spent as dry cow | [www.landbrugsinfo.dk](http://www.landbrugsinfo.dk/) |
| Max Age | 3650 | Maximum age before a cow is culled | Expert opinion |
| Price ECM | 0.313 | The income (EUR) of selling 1 kg ECM (mean of high and low milk price) | (Aes, 2009) |
| Feed Unit Cost | 0.133 | The cost (EUR) for one feed unit (Roughage) | (Aes, 2009) |
| EUR per Hour | 16 | The cost (EUR) for one hour labor | (Kudahl et al., 2011) |
| Insemination Price | 16.1 | The cost (EUR) for one insemination | (Kudahl et al., 2007b) |
| Destruction Price | 64.8 | The cost (EUR) for destroying one animal (Excl. VAT) | [www.daka.dk](http://www.daka.dk/) (07/08/2014) |
| ELISA price | 5.3 | The cost (EUR) of one ELISA | (Kudahl et al., 2007b) |

**Table 4.** MAP-related parameters used in the simulation model. The columns show each parameter, the default value used in the simulations, an explanation of the implementation of the parameter, and the reference for the chosen value. *N* (*x, sd*) describes a normal distribution around the mean *x* with the standard deviation *sd*. Parameters names and default values used in the iCull model are described and explained. References are given in the right column

| Parameter | Default value | Explanation | Reference |
| --- | --- | --- | --- |
| Number of Farming machines | 1 | Number of machines used in all farm sections |  |
| Number of Personnel | 2 | Number of personnel working in all farm sections |  |
| Machine cross-contamination | 8% | The amount of MAP shed within each farm section that is spread with machines | Expert opinion |
| Boot cross-contamination | 1% | The amount of MAP shed within each farm section that is spread with boots | Expert opinion |
| Force of Infection | 0.00016 | The force of infection from environmental MAP (scaling parameter) | Calibrated in the model |
| Hygiene Level | 1 | The hygiene level on the farm. Lower hygiene level will increase the infection pressure from environmental MAP (scaling parameter) | Default set to 1 |
| Low Shedding | 5% | Level of shedding from infected and low shedding animals | Expert opinion |
| High Shedding | 20% | Level of shedding from infected and high shedding animals | Expert opinion |
| Affected Shedding | 100% | Level of shedding from infected and affected animals | Expert opinion |
| Colostrum Risk | 2% | Daily risk of calf getting infected from colostrum from if not pasteurized (per calving) | Calibrated in the model |
| Wastemilk Risk | 0.32% | Daily risk of calf getting infected from wastemilk if not pasteurized (per calving) | Calibrated in the model |
| Calf Risk 1 | 9% | Risk of calf getting infected from infected and low shedding mother (per calving) | (Whittington and Windsor., 2009) |
| Calf Risk 2 | 9% | Risk of calf getting infected from infected and high shedding mother (per calving) | (Whittington and Windsor., 2009) |
| Calf Risk 3 | 39% | Risk of calf getting infected from infected and affected mother (per calving) | (Whittington and Windsor, 2009) |
| Days State 3 | *N* (1095*,* 109*.*5) | Number of days spent in the affected state if not culled. Affected cows are rendered after dying from disease | Expert opinion |
| Days State 2 | *N* (365*,* 36*.*5) | Number of days spent in the high shed- ding state before being affected | Expert opinion |
| Days State 1 | *N* (180*,* 18) | Number of days spent in the low shed- ding state before progressing to the high shedding state | Expert opinion |
| Cutoff | *≥* 0.3 | Cutoff used to identify a positive ELISA | ID Screen (IDvet, Graebels, France) |

**Table 5.** Initial properties of the simulated herd in the iCull model, showing the distribution of animals in each life stage in the initial herd. Par columns show the percentages of cows in parity 1, 2 and 3+, for each of the life steps. The initial herd is constructed to reflect a medium sized dairy herd with 200 cows.

| Parameter | Distribution | No. animals | Par 1 | Par 2 | Par 3+ |
| --- | --- | --- | --- | --- | --- |
| Calves | 27% | 118 |  |  |  |
| Heifers | 10% | 44 |  |  |  |
| Inseminated heifers | 1% | 4 |  |  |  |
| Pregnant heifers | 18% | 79 |  |  |  |
| Early lactation cows | 7% | 30 | 57% | 30% | 13% |
| Inseminated cows | 7% | 30 | 30% | 43% | 27% |
| Pregnant cows | 25% | 110 | 43% | 26% | 31% |
| Dry cows | 7% | 30 | 53% | 23% | 24% |

**Table 6.** Mortalities and stillbirth rates used in the model

| Parameter | Default value | Explanation | Reference |
| --- | --- | --- | --- |
| death0 | 0.065 | Yearly mortality for calves | Expert opinion |
| death1 | 0.035 | Yearly mortality for heifers | Estimated to be between 3.2 and 3.7% (SEGES 2015) |
| death2 | 0.05 | Yearly mortality for inseminated heifers | Expert opinion |
| death3 | 0.05 | Yearly mortality for pregnant heifers | Expert opinion |
| death4 | 0.05 | Yearly mortality for milking cows | Expert opinion |
| death5 | 0.05 | Yearly mortality for inseminated cows | Expert opinion |
| death6 | 0.05 | Yearly mortality for pregnant cows | Expert opinion |
| death7 | 0.05 | Yearly mortality for dry cows | Expert opinion |
| Stillbirth | 0.04 | Risk of stillbirth per calving | Expert opinion |

**Table 7.** Epidemiological results of the sensitivity analysis covering 500 simulations of 10 years each. TP = True prevalence, AP = Apparent prevalence. Numbers show the percentiles. Start prevalence = 5.6%. Cull modulus describes how often the farmer makes culling decisions. Max. heat cycles describes how many cycles a cow can have before it is marked for culling. Voluntary culling is the proportion of cullings that are voluntary. Heat detection describes the proportion of heats that are detected. Insemination success describes the proportion of successful inseminations. Price ECM describes the price for 1 kg ECM milk. EUR per hour labor is the hourly wage for farm personnel. Insemination price is the total price for an insemination. Destruction price is the total price for destruction of an animal. Elisa Price is the total cost for an ELISA test. Number of machines and personnel are those who operate daily in all farm compartments. Boot and machine cross-contamination are the daily proportions of cross-contamination. Low-shedding amount is the amount of MAP shed from animals in the low-shedding stage.

| Parameter | Value | TP 5% | TP 50% | TP 95% | AP 5% | AP 50% | AP 95% |
| --- | --- | --- | --- | --- | --- | --- | --- |
| Cull modulus | 1 | 0.00 | 6.90 | 18.72 | 1.45 | 5.91 | 14.29 |
| Cull modulus | 3 | 0.00 | 6.40 | 16.28 | 1.44 | 5.91 | 13.24 |
| Cull modulus | 7 | 0.00 | 7.02 | 18.46 | 1.46 | 5.91 | 13.81 |
| Cull modulus | 10 | 0.49 | 7.30 | 18.63 | 1.47 | 6.27 | 14.52 |
| Cull modulus | 13 | 0.49 | 7.39 | 17.73 | 1.48 | 6.16 | 13.33 |
| max. heat cycles | 3 | 0.00 | 1.94 | 8.39 | 0.49 | 2.45 | 6.90 |
| max. heat cycles | 5 | 0.00 | 5.37 | 14.15 | 0.99 | 4.90 | 11.11 |
| max. heat cycles | 7 | 0.00 | 7.02 | 18.46 | 1.46 | 5.91 | 13.81 |
| max. heat cycles | 10 | 0.00 | 7.32 | 18.32 | 1.48 | 6.31 | 14.22 |
| max. heat cycles | 13 | 0.48 | 7.79 | 18.63 | 1.47 | 6.37 | 14.71 |
| Frac. voluntary culling | 0.1 | 0.00 | 2.46 | 10.84 | 0.49 | 2.96 | 8.29 |
| Frac. voluntary culling | 0.33 | 0.00 | 7.02 | 18.46 | 1.46 | 5.91 | 13.81 |
| Frac. voluntary culling | 0.5 | 2.95 | 13.53 | 26.84 | 2.95 | 10.65 | 20.30 |
| Frac. voluntary culling | 0.7 | 11.81 | 28.57 | 42.94 | 9.31 | 22.06 | 32.35 |
| Heat detection -cow | 0.26 | 0.00 | 7.02 | 18.46 | 1.46 | 5.91 | 13.81 |
| Heat detection -cow | 0.36 | 0.00 | 7.02 | 18.46 | 1.46 | 5.91 | 13.81 |
| Heat detection -cow | 0.46 | 0.00 | 7.02 | 18.46 | 1.46 | 5.91 | 13.81 |
| Heat detection -heifer | 0.5 | 1.46 | 8.70 | 19.71 | 1.96 | 7.32 | 15.05 |
| Heat detection -heifer | 0.6 | 0.00 | 7.02 | 18.46 | 1.46 | 5.91 | 13.81 |
| Heat detection -heifer | 0.7 | 0.00 | 6.34 | 16.26 | 1.46 | 5.83 | 12.63 |
| Insem. succes -cow | 0.32 | 0.00 | 6.91 | 17.66 | 1.46 | 5.88 | 14.37 |
| Insem. succes -cow | 0.42 | 0.00 | 7.02 | 18.46 | 1.46 | 5.91 | 13.81 |
| Insem. succes -cow | 0.52 | 0.00 | 6.85 | 15.69 | 0.99 | 6.07 | 11.92 |
| Insem. succes -heifer | 0.45 | 0.00 | 7.09 | 18.45 | 1.46 | 5.91 | 13.73 |
| Insem. succes -heifer | 0.55 | 0.00 | 7.02 | 18.46 | 1.46 | 5.91 | 13.81 |
| Insem. succes -heifer | 0.65 | 0.00 | 6.86 | 17.07 | 1.45 | 5.88 | 13.24 |
| Price ECM | 0.374 | 0.00 | 7.02 | 18.46 | 1.46 | 5.91 | 13.81 |
| Price ECM | 0.384 | 0.00 | 7.02 | 18.46 | 1.46 | 5.91 | 13.81 |
| Price ECM | 0.394 | 0.00 | 7.02 | 18.46 | 1.46 | 5.91 | 13.81 |
| Euro per hour labour | 15 | 0.00 | 7.02 | 18.46 | 1.46 | 5.91 | 13.81 |
| Euro per hour labour | 25 | 0.00 | 7.02 | 18.46 | 1.46 | 5.91 | 13.81 |
| Euro per hour labour | 35 | 0.00 | 7.02 | 18.46 | 1.46 | 5.91 | 13.81 |
| Insemination price | 8.33 | 0.00 | 7.02 | 18.46 | 1.46 | 5.91 | 13.81 |
| Insemination price | 13.33 | 0.00 | 7.02 | 18.46 | 1.46 | 5.91 | 13.81 |
| Insemination price | 18.33 | 0.00 | 7.02 | 18.46 | 1.46 | 5.91 | 13.81 |
| Destruction price | 69 | 0.00 | 7.02 | 18.46 | 1.46 | 5.91 | 13.81 |
| Destruction price | 79 | 0.00 | 7.02 | 18.46 | 1.46 | 5.91 | 13.81 |
| Destruction price | 89 | 0.00 | 7.02 | 18.46 | 1.46 | 5.91 | 13.81 |
| Elisa price | 3.29 | 0.00 | 7.02 | 18.46 | 1.46 | 5.91 | 13.81 |
| Elisa price | 3.79 | 0.00 | 7.02 | 18.46 | 1.46 | 5.91 | 13.81 |
| Elisa price | 4.29 | 0.00 | 7.02 | 18.46 | 1.46 | 5.91 | 13.81 |
| Number of machines | 1 | 0.00 | 7.02 | 18.46 | 1.46 | 5.91 | 13.81 |
| Number of machines | 2 | 0.00 | 6.58 | 17.57 | 1.43 | 5.85 | 13.37 |
| Number of machines | 4 | 0.00 | 6.31 | 16.99 | 0.99 | 5.61 | 13.11 |
| Number of personnel | 2 | 0.00 | 7.02 | 18.46 | 1.46 | 5.91 | 13.81 |
| Number of personnel | 3 | 0.00 | 6.90 | 18.63 | 1.46 | 5.91 | 13.31 |
| Number of personnel | 4 | 0.00 | 6.88 | 17.88 | 1.47 | 5.91 | 12.99 |
| Number of personnel | 6 | 0.00 | 6.83 | 17.75 | 1.46 | 5.91 | 13.66 |
| Boot cross-contamination | 0.01 | 0.48 | 7.77 | 19.43 | 1.47 | 6.34 | 15.61 |
| Boot cross-contamination | 0.1 | 0.00 | 6.39 | 17.57 | 0.99 | 5.64 | 13.31 |
| Boot cross-contamination | 0.2 | 0.00 | 5.39 | 15.28 | 0.98 | 4.88 | 12.14 |
| Boot cross-contamination | 0.4 | 0.00 | 3.41 | 11.11 | 0.49 | 3.45 | 8.88 |
| Boot cross-contamination | 0.6 | 0.00 | 1.95 | 8.74 | 0.49 | 2.90 | 7.32 |
| Machine cross-contamination | 0.04 | 0.49 | 7.34 | 19.13 | 1.48 | 6.31 | 15.61 |
| Machine cross-contamination | 0.08 | 0.00 | 6.90 | 18.46 | 1.47 | 5.88 | 13.79 |
| Machine cross-contamination | 0.16 | 0.00 | 6.58 | 17.88 | 0.99 | 5.84 | 13.53 |
| Machine cross-contamination | 0.32 | 0.00 | 5.39 | 15.20 | 0.98 | 4.89 | 12.08 |
| Low-shedding amount | 0.01 | 0.00 | 1.96 | 8.25 | 0.49 | 2.91 | 7.25 |
| Low-shedding amount | 0.05 | 0.00 | 7.02 | 18.46 | 1.46 | 5.91 | 13.81 |
| Low-shedding amount | 0.1 | 8.24 | 26.21 | 41.67 | 6.37 | 18.58 | 29.62 |
| Low-shedding amount | 0.2 | 60.57 | 67.32 | 73.66 | 39.02 | 45.63 | 51.22 |

**Table 8.** Epidemiological results of the sensitivity analysis covering 500 simulations of 10 years each. TP = True prevalence, AP = Apparent prevalence. Numbers show the percentiles. Start prevalence = 5.6%. Low, High and Affected-shedding amount is the amount of shedding for an animal in each stage of disease. Vertical transmission rates are also described for each disease state. Discard milk is the number of days the farmer discards milk at the beginning of each lactation. Risk stillbirth is the risk of stillbirth per calving. Durations for the low-shedding, high-shedding and affected stage are described. Hygiene level describes how likely it is that MAP will be cleaned out from each farm compartment. Test specificity is the specificity for the ELISA test used in the model. Sensitivity level is a vertical adjustment factor for the age-dependent sensitivity function used in the model.

| Parameter | Value | TP 5% | TP 50% | TP 95% | AP 5% | AP 50% | AP 95% |
| --- | --- | --- | --- | --- | --- | --- | --- |
| High-shedding amount | 0.2 | 0.00 | 7.02 | 18.46 | 1.46 | 5.91 | 13.81 |
| High-shedding amount | 0.3 | 0.49 | 10.78 | 22.66 | 1.48 | 8.72 | 16.84 |
| High-shedding amount | 0.4 | 1.97 | 14.67 | 27.10 | 2.43 | 10.84 | 19.91 |
| Affected-shedding amount | 0.4 | 0.00 | 1.96 | 7.81 | 0.48 | 2.90 | 6.98 |
| Affected-shedding amount | 0.5 | 0.00 | 2.44 | 8.87 | 0.49 | 2.94 | 7.41 |
| Affected-shedding amount | 0.6 | 0.00 | 2.94 | 11.28 | 0.49 | 3.43 | 8.93 |
| Affected-shedding amount | 0.7 | 0.00 | 4.14 | 13.24 | 0.97 | 3.92 | 10.73 |
| Affected-shedding amount | 0.8 | 0.00 | 4.88 | 14.08 | 0.98 | 4.88 | 11.27 |
| Affected-shedding amount | 1 | 0.00 | 7.02 | 18.46 | 1.46 | 5.91 | 13.81 |
| Low-shedding vert. trans. | 0.04 | 0.00 | 6.73 | 16.02 | 0.99 | 5.85 | 12.62 |
| Low-shedding vert. transm. | 0.09 | 0.00 | 7.02 | 18.46 | 1.46 | 5.91 | 13.81 |
| Low-shedding vert. transm. | 0.15 | 0.49 | 8.25 | 19.42 | 1.47 | 6.86 | 14.22 |
| High-shedding vert. transm. | 0.19 | 0.00 | 7.28 | 18.84 | 1.46 | 5.91 | 13.80 |
| High-shedding vert. transm. | 0.39 | 0.00 | 7.35 | 20.49 | 1.46 | 6.34 | 15.12 |
| High-shedding vert. transm. | 0.59 | 0.49 | 8.23 | 19.71 | 1.46 | 6.75 | 14.82 |
| Affected vertical transm. | 0.1 | 0.00 | 6.80 | 18.85 | 1.46 | 5.90 | 14.17 |
| Affected vertical transm. | 0.5 | 0.00 | 6.86 | 17.57 | 1.48 | 5.88 | 13.66 |
| Affected vertical transm. | 0.8 | 0.00 | 6.90 | 17.73 | 1.47 | 5.90 | 13.53 |
| Discard Milk (days) | 2 | 0.00 | 7.02 | 18.46 | 1.46 | 5.91 | 13.81 |
| Discard Milk (days) | 4 | 0.00 | 6.39 | 17.17 | 0.98 | 5.85 | 12.81 |
| Discard Milk (days) | 6 | 0.00 | 6.31 | 16.27 | 0.98 | 5.83 | 13.11 |
| Risk stillbirth | 0.02 | 0.00 | 6.31 | 16.10 | 0.98 | 5.37 | 12.62 |
| Risk stillbirth | 0.04 | 0.00 | 7.02 | 18.46 | 1.46 | 5.91 | 13.81 |
| Risk stillbirth | 0.08 | 0.97 | 8.49 | 19.42 | 1.46 | 6.88 | 15.05 |
| Risk stillbirth | 0.14 | 0.97 | 10.29 | 24.02 | 1.93 | 8.37 | 17.57 |
| Days low-shedding | 730 | 3.43 | 18.54 | 35.62 | 3.40 | 13.69 | 25.17 |
| Days low-shedding | 912 | 0.45 | 10.84 | 24.39 | 1.47 | 8.82 | 18.05 |
| Days low-shedding | 1095 | 0.00 | 7.02 | 18.46 | 1.46 | 5.91 | 13.81 |
| Days low-shedding | 1277 | 0.00 | 4.88 | 12.88 | 0.98 | 4.43 | 10.30 |
| Days low-shedding | 1460 | 0.00 | 3.42 | 11.22 | 0.97 | 3.88 | 9.31 |
| Days high-shedding | 182 | 0.49 | 7.37 | 17.97 | 1.46 | 6.34 | 13.66 |
| Days high-shedding | 365 | 0.00 | 7.02 | 18.46 | 1.46 | 5.91 | 13.81 |
| Days high-shedding | 548 | 0.00 | 6.93 | 18.05 | 0.98 | 6.10 | 13.24 |
| Days affected | 45 | 0.00 | 0.99 | 6.76 | 0.49 | 2.42 | 6.37 |
| Days affected | 91 | 0.00 | 2.46 | 10.73 | 0.49 | 3.37 | 8.78 |
| Days affected | 182 | 0.00 | 6.88 | 18.65 | 0.98 | 5.91 | 14.64 |
| Days affected | 225 | 0.49 | 9.76 | 22.67 | 1.47 | 7.80 | 17.32 |
| Days affected | 271 | 1.47 | 11.68 | 24.64 | 1.97 | 9.38 | 17.96 |
| Hygiene level | 1.2 | 0.00 | 2.96 | 11.29 | 0.49 | 3.43 | 8.34 |
| Hygiene level | 1.1 | 0.00 | 4.88 | 14.71 | 0.97 | 4.39 | 11.78 |
| Hygiene level | 1.0 | 0.00 | 7.02 | 18.46 | 1.46 | 5.91 | 13.81 |
| Hygiene level | 0.9 | 0.98 | 10.84 | 24.15 | 1.92 | 8.37 | 18.23 |
| Hygiene level | 0.8 | 2.93 | 15.61 | 30.20 | 3.41 | 11.65 | 21.67 |
| Hygiene level | 0.7 | 9.22 | 25.79 | 40.78 | 7.30 | 18.72 | 28.61 |
| Hygiene level | 0.6 | 19.79 | 38.73 | 52.91 | 14.84 | 27.07 | 37.68 |
| Hygiene level | 0.5 | 36.70 | 55.61 | 66.50 | 24.87 | 37.75 | 46.08 |
| Test specificity | 70 | 0.00 | 6.39 | 17.08 | 26.83 | 33.01 | 38.92 |
| Test specificity | 80 | 0.00 | 6.90 | 16.91 | 17.07 | 23.04 | 30.54 |
| Test specificity | 90 | 0.00 | 6.80 | 17.65 | 8.29 | 13.66 | 20.98 |
| Test specificity | 95 | 0.49 | 7.32 | 16.59 | 4.41 | 9.71 | 16.03 |
| Test specificity | 98.67 | 0.00 | 7.02 | 18.46 | 1.46 | 5.91 | 13.81 |
| Test specificity | 99 | 0.00 | 6.37 | 17.07 | 0.98 | 5.39 | 13.11 |
| Test specificity | 99.5 | 0.00 | 7.77 | 17.48 | 0.49 | 5.39 | 12.56 |
| Sensitivity level | 0.5 | 0.00 | 7.28 | 19.02 | 0.97 | 3.84 | 8.29 |
| Sensitivity level | 0.6 | 0.00 | 7.28 | 17.08 | 0.98 | 3.95 | 8.29 |
| Sensitivity level | 0.7 | 0.00 | 7.39 | 18.24 | 0.98 | 4.43 | 10.24 |
| Sensitivity level | 0.8 | 0.49 | 7.37 | 18.07 | 1.46 | 5.34 | 11.71 |
| Sensitivity level | 0.9 | 0.00 | 6.86 | 17.33 | 1.46 | 5.85 | 12.69 |
| Sensitivity level | 1 | 0.00 | 7.02 | 18.46 | 1.46 | 5.91 | 13.81 |
| Sensitivity level | 1.1 | 0.00 | 6.90 | 16.67 | 0.99 | 6.70 | 14.15 |
| Sensitivity level | 1.2 | 0.00 | 6.83 | 16.35 | 1.46 | 6.40 | 14.57 |

**Table 9.** Epidemiological results of the sensitivity analysis covering 500 simulations of 10 years each. TP = True prevalence, AP = Apparent prevalence. Numbers show the percentiles. Start prevalence = 5.6%. Prop. showing fast heat describes the proportion of cows that are not successfully inseminated and can be inseminated again after 21 days. Frac. low producing cows for cull.list describes the fraction of the lowest producing cows that the farmer flags for the culling list in the model. Days in calving box is the number of days spent in the calving box when calving. Feed unit cost is the cost of one feed unit. Wastemilk infection and colostrum risk are the daily risks of being infected from each source. Risk reduction when removing calves describes the reduction in risk of infection from the dam when the calf is removed within 2 hours after birth.

| Parameter | Value | TP 5% | TP 50% | TP 95% | AP 5% | AP 50% | AP 95% |
| --- | --- | --- | --- | --- | --- | --- | --- |
| Prop. showing fast heat | 0.5 | 0.00 | 7.02 | 18.46 | 1.46 | 5.91 | 13.81 |
| Prop. showing fast heat | 0.1 | 0.00 | 7.02 | 18.46 | 1.46 | 5.91 | 13.81 |
| Prop. showing fast heat | 0.15 | 0.00 | 7.02 | 18.46 | 1.46 | 5.91 | 13.81 |
| Prop. showing fast heat | 0.25 | 0.00 | 7.02 | 18.46 | 1.46 | 5.91 | 13.81 |
| Prop. showing fast heat | 0.50 | 0.00 | 7.02 | 18.46 | 1.46 | 5.91 | 13.81 |
| Frac. low prod. cows for cull.list | 0.1 | 0.00 | 4.85 | 14.64 | 0.97 | 4.41 | 11.22 |
| Frac. low prod. cows for cull.list | 0.2 | 0.00 | 7.02 | 18.46 | 1.46 | 5.91 | 13.81 |
| Frac. low prod. cows for cull.list | 0.5 | 0.00 | 1.47 | 7.81 | 0.49 | 2.44 | 6.83 |
| Frac. low prod. cows for cull.list | 0.8 | 0.00 | 0.00 | 3.48 | 0.48 | 1.47 | 3.45 |
| Frac. low prod. cows for cull.list | 0 | 0.00 | 7.18 | 18.46 | 1.46 | 5.91 | 13.81 |
| Frac. low prod. cows for cull.list | 100 | 0.00 | 7.02 | 18.46 | 1.46 | 5.91 | 13.81 |
| Frac. low prod. cows for cull.list | 200 | 0.00 | 7.02 | 18.46 | 1.46 | 5.91 | 13.81 |
| Frac. low prod. cows for cull.list | 300 | 0.00 | 7.02 | 18.46 | 1.46 | 5.91 | 13.81 |
| Days in calving box | 1 | 0.00 | 6.90 | 18.46 | 1.46 | 5.91 | 13.81 |
| Days in calving box | 3 | 0.00 | 7.02 | 18.46 | 1.46 | 5.91 | 13.81 |
| Days in calving box | 5 | 0.00 | 7.02 | 18.46 | 1.46 | 5.91 | 13.81 |
| Days in calving box | 10 | 0.00 | 7.02 | 18.46 | 1.46 | 5.91 | 13.81 |
| Feed unit cost | 0.100 | 0.00 | 7.02 | 18.46 | 1.46 | 5.91 | 13.81 |
| Feed unit cost | 0.133 | 0.00 | 7.02 | 18.46 | 1.46 | 5.91 | 13.81 |
| Feed unit cost | 2.00 | 0.00 | 7.02 | 18.46 | 1.46 | 5.91 | 13.81 |
| Feed unit cost | 5.00 | 0.00 | 7.02 | 18.46 | 1.46 | 5.91 | 13.81 |
| Wastemilk infection risk | 0.0022 | 0.00 | 4.87 | 12.75 | 0.99 | 4.41 | 10.85 |
| Wastemilk infection risk | 0.0032 | 0.00 | 4.87 | 12.75 | 0.99 | 4.41 | 10.85 |
| Wastemilk infection risk | 0.0042 | 0.00 | 4.87 | 12.75 | 0.99 | 4.41 | 10.85 |
| Colostrum infection risk | 0.01 | 0.00 | 5.33 | 16.02 | 0.98 | 4.87 | 12.68 |
| Colostrum infection risk | 0.02 | 0.00 | 5.33 | 16.02 | 0.98 | 4.87 | 12.68 |
| Colostrum infection risk | 0.05 | 0.00 | 5.33 | 16.02 | 0.98 | 4.87 | 12.68 |
| Risk reduction when removing calves | 0.00 | 0.49 | 7.90 | 19.12 | 1.48 | 6.73 | 14.22 |
| Risk reduction when removing calves | 0.50 | 0.00 | 6.36 | 17.57 | 1.45 | 5.37 | 13.24 |
| Risk reduction when removing calves | 0.95 | 0.00 | 5.91 | 15.20 | 1.42 | 5.37 | 12.25 |
| Risk reduction when removing calves | 1.00 | 0.00 | 5.81 | 15.39 | 0.98 | 4.93 | 12.20 |

**Table 10.** Production and Economic results of the sensitivity analysis. Numbers are in million KG or million EUR. ECM = Milk yield, Exp = expenses, Inc = income, TNR = Total net revenue over ten years. Numbers describe the percentiles of 500 repetitions of 10 simulation years. Start prevalence = 5.6%. Cull modulus describes how often the farmer makes culling decisions. Max. heat cycles describes how many cycles a cow can have before it is marked for culling. Voluntary culling is the proportion of cullings that are voluntary. Heat detection describes the proportion of heats that are detected. Insemination success describes the proportion of successful inseminations. Price ECM describes the price for 1 kg ECM milk. EUR per hour labor is the hourly wage for farm personnel. Insemination price is the total price for an insemination. Destruction price is the total price for destruction of an animal. Elisa Price is the total cost for an ELISA test. Number of machines and personnel are those who operate daily in all farm compartments. Boot and machine cross-contamination are the daily proportions of cross-contamination. Low-shedding amount is the amount of MAP shed from animals in the low-shedding stage.

| Parameter | Value | ECM 5% | ECM 50% | ECM 95% | Exp. 5% | Exp. 50% | Exp. 95% | Inc. 5% | Inc. 50% | Inc. 95% | Median TNR |
| --- | --- | --- | --- | --- | --- | --- | --- | --- | --- | --- | --- |
| Cull modulus | 1 | 19.79 | 20.03 | 20.29 | 5.04 | 5.11 | 5.17 | 7.96 | 8.08 | 8.22 | 2.98 |
| Cull modulus | 3 | 19.81 | 20.05 | 20.34 | 5.05 | 5.11 | 5.18 | 7.96 | 8.09 | 8.22 | 2.98 |
| Cull modulus | 7 | 19.90 | 20.12 | 20.39 | 5.07 | 5.13 | 5.19 | 7.99 | 8.12 | 8.25 | 2.99 |
| Cull modulus | 10 | 19.89 | 20.16 | 20.44 | 5.07 | 5.14 | 5.20 | 7.99 | 8.13 | 8.25 | 3.00 |
| Cull modulus | 13 | 19.98 | 20.23 | 20.47 | 5.09 | 5.16 | 5.22 | 8.05 | 8.16 | 8.29 | 3.01 |
| max. heat cycles | 3 | 19.92 | 20.14 | 20.39 | 5.01 | 5.07 | 5.13 | 8.08 | 8.19 | 8.30 | 3.13 |
| max. heat cycles | 5 | 19.90 | 20.15 | 20.41 | 5.05 | 5.12 | 5.18 | 8.01 | 8.15 | 8.27 | 3.03 |
| max. heat cycles | 7 | 19.90 | 20.12 | 20.39 | 5.07 | 5.13 | 5.19 | 7.99 | 8.12 | 8.25 | 2.99 |
| max. heat cycles | 10 | 19.84 | 20.11 | 20.37 | 5.07 | 5.13 | 5.19 | 7.96 | 8.11 | 8.24 | 2.98 |
| max. heat cycles | 13 | 19.83 | 20.09 | 20.35 | 5.06 | 5.13 | 5.19 | 7.98 | 8.10 | 8.22 | 2.98 |
| Frac. voluntary culling | 0.1 | 19.62 | 19.87 | 20.12 | 4.95 | 5.01 | 5.08 | 7.94 | 8.06 | 8.18 | 3.05 |
| Frac. voluntary culling | 0.33 | 19.90 | 20.12 | 20.39 | 5.07 | 5.13 | 5.19 | 7.99 | 8.12 | 8.25 | 2.99 |
| Frac. voluntary culling | 0.5 | 20.08 | 20.35 | 20.61 | 5.16 | 5.23 | 5.30 | 7.99 | 8.16 | 8.29 | 2.92 |
| Frac. voluntary culling | 0.7 | 20.38 | 20.69 | 20.97 | 5.30 | 5.37 | 5.46 | 7.96 | 8.19 | 8.37 | 2.82 |
| Heat detection -cow | 0.26 | 19.90 | 20.12 | 20.39 | 5.07 | 5.13 | 5.19 | 7.99 | 8.12 | 8.25 | 2.99 |
| Heat detection -cow | 0.36 | 19.90 | 20.12 | 20.39 | 5.07 | 5.13 | 5.19 | 7.99 | 8.12 | 8.25 | 2.99 |
| Heat detection -cow | 0.46 | 19.90 | 20.12 | 20.39 | 5.07 | 5.13 | 5.19 | 7.99 | 8.12 | 8.25 | 2.99 |
| Heat detection -heifer | 0.5 | 19.74 | 19.99 | 20.23 | 5.00 | 5.07 | 5.13 | 7.93 | 8.06 | 8.17 | 2.99 |
| Heat detection -heifer | 0.6 | 19.90 | 20.12 | 20.39 | 5.07 | 5.13 | 5.19 | 7.99 | 8.12 | 8.25 | 2.99 |
| Heat detection -heifer | 0.7 | 19.94 | 20.21 | 20.45 | 5.10 | 5.17 | 5.23 | 8.02 | 8.16 | 8.29 | 2.99 |
| Insem. succes -cow | 0.32 | 19.68 | 19.93 | 20.19 | 4.97 | 5.03 | 5.09 | 7.90 | 8.04 | 8.17 | 3.01 |
| Insem. succes -cow | 0.42 | 19.90 | 20.12 | 20.39 | 5.07 | 5.13 | 5.19 | 7.99 | 8.12 | 8.25 | 2.99 |
| Insem. succes -cow | 0.52 | 20.00 | 20.24 | 20.48 | 5.13 | 5.18 | 5.25 | 8.05 | 8.17 | 8.29 | 2.99 |
| Insem. succes -heifer | 0.45 | 19.87 | 20.13 | 20.38 | 5.09 | 5.15 | 5.22 | 7.98 | 8.13 | 8.25 | 2.96 |
| Insem. succes -heifer | 0.55 | 19.90 | 20.12 | 20.39 | 5.07 | 5.13 | 5.19 | 7.99 | 8.12 | 8.25 | 2.99 |
| Insem. succes -heifer | 0.65 | 19.89 | 20.13 | 20.42 | 5.05 | 5.12 | 5.18 | 7.99 | 8.13 | 8.27 | 3.02 |
| Price ECM | 0.374 | 19.90 | 20.12 | 20.39 | 5.07 | 5.13 | 5.19 | 7.79 | 7.92 | 8.05 | 2.79 |
| Price ECM | 0.384 | 19.90 | 20.12 | 20.39 | 5.07 | 5.13 | 5.19 | 7.99 | 8.12 | 8.25 | 2.99 |
| Price ECM | 0.394 | 19.90 | 20.12 | 20.39 | 5.07 | 5.13 | 5.19 | 8.19 | 8.32 | 8.46 | 3.19 |
| Euro per hour labour | 15 | 19.90 | 20.12 | 20.39 | 5.07 | 5.13 | 5.19 | 7.99 | 8.12 | 8.25 | 2.99 |
| Euro per hour labour | 25 | 19.90 | 20.12 | 20.39 | 5.07 | 5.13 | 5.19 | 7.99 | 8.12 | 8.25 | 2.99 |
| Euro per hour labour | 35 | 19.90 | 20.12 | 20.39 | 5.07 | 5.13 | 5.19 | 7.99 | 8.12 | 8.25 | 2.99 |
| Insemination price | 8.33 | 19.90 | 20.12 | 20.39 | 5.04 | 5.09 | 5.15 | 7.99 | 8.12 | 8.25 | 3.03 |
| Insemination price | 13.33 | 19.90 | 20.12 | 20.39 | 5.07 | 5.13 | 5.19 | 7.99 | 8.12 | 8.25 | 2.99 |
| Insemination price | 18.33 | 19.90 | 20.12 | 20.39 | 5.11 | 5.17 | 5.23 | 7.99 | 8.12 | 8.25 | 2.95 |
| Destruction price | 69 | 19.90 | 20.12 | 20.39 | 5.07 | 5.13 | 5.19 | 7.99 | 8.12 | 8.25 | 2.99 |
| Destruction price | 79 | 19.90 | 20.12 | 20.39 | 5.07 | 5.13 | 5.19 | 7.99 | 8.12 | 8.25 | 2.99 |
| Destruction price | 89 | 19.90 | 20.12 | 20.39 | 5.08 | 5.13 | 5.19 | 7.99 | 8.12 | 8.25 | 2.99 |
| Elisa price | 3.29 | 19.90 | 20.12 | 20.39 | 5.07 | 5.13 | 5.19 | 7.99 | 8.12 | 8.25 | 2.99 |
| Elisa price | 3.79 | 19.90 | 20.12 | 20.39 | 5.07 | 5.13 | 5.19 | 7.99 | 8.12 | 8.25 | 2.99 |
| Elisa price | 4.29 | 19.90 | 20.12 | 20.39 | 5.08 | 5.13 | 5.19 | 7.99 | 8.12 | 8.25 | 2.99 |
| Number of machines | 1 | 19.90 | 20.12 | 20.39 | 5.07 | 5.13 | 5.19 | 7.99 | 8.12 | 8.25 | 2.99 |
| Number of machines | 2 | 19.86 | 20.14 | 20.38 | 5.07 | 5.13 | 5.19 | 7.99 | 8.12 | 8.26 | 2.99 |
| Number of machines | 4 | 19.86 | 20.15 | 20.40 | 5.07 | 5.13 | 5.19 | 8.00 | 8.13 | 8.26 | 3.00 |
| Number of personnel | 2 | 19.90 | 20.12 | 20.39 | 5.07 | 5.13 | 5.19 | 7.99 | 8.12 | 8.25 | 2.99 |
| Number of personnel | 3 | 19.89 | 20.12 | 20.37 | 5.07 | 5.13 | 5.19 | 7.99 | 8.12 | 8.26 | 2.99 |
| Number of personnel | 4 | 19.86 | 20.12 | 20.37 | 5.07 | 5.13 | 5.19 | 7.99 | 8.12 | 8.25 | 2.99 |
| Number of personnel | 6 | 19.86 | 20.12 | 20.37 | 5.07 | 5.13 | 5.19 | 7.99 | 8.12 | 8.25 | 2.99 |
| Boot cross-contamination | 0.01 | 19.88 | 20.12 | 20.38 | 5.06 | 5.13 | 5.20 | 7.99 | 8.12 | 8.25 | 2.99 |
| Boot cross-contamination | 0.1 | 19.85 | 20.15 | 20.40 | 5.07 | 5.13 | 5.20 | 7.99 | 8.12 | 8.26 | 2.99 |
| Boot cross-contamination | 0.2 | 19.91 | 20.15 | 20.38 | 5.07 | 5.13 | 5.19 | 8.01 | 8.13 | 8.25 | 3.00 |
| Boot cross-contamination | 0.4 | 19.88 | 20.14 | 20.39 | 5.07 | 5.13 | 5.19 | 7.99 | 8.12 | 8.26 | 2.99 |
| Boot cross-contamination | 0.6 | 19.89 | 20.16 | 20.40 | 5.08 | 5.13 | 5.19 | 7.99 | 8.13 | 8.26 | 3.00 |
| Machine cross-contamination | 0.04 | 19.87 | 20.12 | 20.39 | 5.07 | 5.13 | 5.19 | 7.98 | 8.11 | 8.25 | 2.99 |
| Machine cross-contamination | 0.08 | 19.89 | 20.13 | 20.39 | 5.07 | 5.13 | 5.19 | 8.00 | 8.12 | 8.26 | 2.99 |
| Machine cross-contamination | 0.16 | 19.86 | 20.14 | 20.38 | 5.07 | 5.13 | 5.19 | 7.99 | 8.12 | 8.26 | 2.99 |
| Machine cross-contamination | 0.32 | 19.90 | 20.14 | 20.38 | 5.07 | 5.13 | 5.19 | 8.00 | 8.13 | 8.25 | 3.00 |
| Low-shedding amount | 0.01 | 19.93 | 20.17 | 20.41 | 5.07 | 5.14 | 5.20 | 8.00 | 8.14 | 8.26 | 3.00 |
| Low-shedding amount | 0.05 | 19.90 | 20.12 | 20.39 | 5.07 | 5.13 | 5.19 | 7.99 | 8.12 | 8.25 | 2.99 |
| Low-shedding amount | 0.1 | 19.80 | 20.04 | 20.29 | 5.05 | 5.11 | 5.18 | 7.95 | 8.08 | 8.21 | 2.97 |
| Low-shedding amount | 0.2 | 19.48 | 19.71 | 19.97 | 4.99 | 5.05 | 5.11 | 7.82 | 7.95 | 8.07 | 2.91 |

**Table 11.** Production and Economic results of the sensitivity analysis. Numbers are in million KG or million EUR. ECM = Milk yield, Exp = expenses, Inc = income, TNR = Total net revenue over ten years. Numbers describe the percentiles of 500 repetitions of 10 simulation years. Start prevalence = 5.6%. Low, High and Affected-shedding amount is the amount of shedding for an animal in each stage of disease. Vertical transmission rates are also described for each disease state. Discard milk is the number of days the farmer discards milk at the beginning of each lactation. Risk stillbirth is the risk of stillbirth per calving. Durations for the low-shedding, high-shedding and affected stage are described. Hygiene level describes how likely it is that MAP will be cleaned out from each farm compartment. Test specificity is the specificity for the ELISA test used in the model. Sensitivity level is a vertical adjustment factor for the age-dependent sensitivity function used in the model.

| Parameter | Value | ECM 5% | ECM 50% | ECM 95% | Exp. 5% | Exp. 50% | Exp. 95% | Inc. 5% | Inc. 50% | Inc. 95% | MedianTNR |
| --- | --- | --- | --- | --- | --- | --- | --- | --- | --- | --- | --- |
| High-shedding amount | 0.2 | 19.90 | 20.12 | 20.39 | 5.07 | 5.13 | 5.19 | 7.99 | 8.12 | 8.25 | 2.99 |
| High-shedding amount | 0.3 | 19.85 | 20.12 | 20.35 | 5.06 | 5.13 | 5.19 | 7.98 | 8.12 | 8.24 | 2.99 |
| High-shedding amount | 0.4 | 19.83 | 20.11 | 20.34 | 5.05 | 5.12 | 5.19 | 7.97 | 8.11 | 8.23 | 2.99 |
| Affected-shedding amount | 0.4 | 19.91 | 20.14 | 20.41 | 5.07 | 5.13 | 5.20 | 8.01 | 8.13 | 8.26 | 3.00 |
| Affected-shedding amount | 0.5 | 19.91 | 20.15 | 20.43 | 5.08 | 5.13 | 5.20 | 8.00 | 8.13 | 8.28 | 3.00 |
| Affected-shedding amount | 0.6 | 19.89 | 20.13 | 20.38 | 5.07 | 5.13 | 5.19 | 8.00 | 8.13 | 8.24 | 3.00 |
| Affected-shedding amount | 0.7 | 19.91 | 20.16 | 20.42 | 5.08 | 5.14 | 5.20 | 8.01 | 8.13 | 8.26 | 3.00 |
| Affected-shedding amount | 0.8 | 19.91 | 20.15 | 20.38 | 5.07 | 5.13 | 5.19 | 8.00 | 8.13 | 8.24 | 2.99 |
| Affected-shedding amount | 1 | 19.90 | 20.12 | 20.39 | 5.07 | 5.13 | 5.19 | 7.99 | 8.12 | 8.25 | 2.99 |
| Low-shedding vert. trans. | 0.04 | 19.87 | 20.13 | 20.38 | 5.07 | 5.13 | 5.20 | 7.99 | 8.13 | 8.25 | 3.00 |
| Low-shedding vert. transm. | 0.09 | 19.90 | 20.12 | 20.39 | 5.07 | 5.13 | 5.19 | 7.99 | 8.12 | 8.25 | 2.99 |
| Low-shedding vert. transm. | 0.15 | 19.88 | 20.12 | 20.38 | 5.07 | 5.13 | 5.19 | 8.00 | 8.12 | 8.25 | 2.99 |
| High-shedding vert. transm. | 0.19 | 19.89 | 20.12 | 20.38 | 5.08 | 5.13 | 5.19 | 7.99 | 8.12 | 8.25 | 2.99 |
| High-shedding vert. transm. | 0.39 | 19.87 | 20.12 | 20.39 | 5.07 | 5.13 | 5.19 | 7.99 | 8.12 | 8.24 | 2.99 |
| High-shedding vert. transm. | 0.59 | 19.85 | 20.11 | 20.39 | 5.07 | 5.13 | 5.19 | 7.98 | 8.12 | 8.25 | 2.99 |
| Affected vertical transm. | 0.1 | 19.90 | 20.12 | 20.40 | 5.07 | 5.13 | 5.19 | 7.99 | 8.11 | 8.27 | 2.99 |
| Affected vertical transm. | 0.5 | 19.90 | 20.13 | 20.39 | 5.07 | 5.13 | 5.19 | 7.99 | 8.12 | 8.25 | 2.99 |
| Affected vertical transm. | 0.8 | 19.88 | 20.12 | 20.41 | 5.07 | 5.13 | 5.20 | 7.98 | 8.12 | 8.25 | 2.99 |
| Discard Milk (days) | 2 | 19.90 | 20.12 | 20.39 | 5.07 | 5.13 | 5.19 | 7.99 | 8.12 | 8.25 | 2.99 |
| Discard Milk (days) | 4 | 19.71 | 19.98 | 20.24 | 5.03 | 5.10 | 5.17 | 7.95 | 8.08 | 8.20 | 2.98 |
| Discard Milk (days) | 6 | 19.55 | 19.81 | 20.05 | 5.00 | 5.07 | 5.13 | 7.90 | 8.02 | 8.15 | 2.96 |
| Risk stillbirth | 0.02 | 19.87 | 20.11 | 20.39 | 5.08 | 5.14 | 5.21 | 8.00 | 8.13 | 8.26 | 2.99 |
| Risk stillbirth | 0.04 | 19.90 | 20.12 | 20.39 | 5.07 | 5.13 | 5.19 | 7.99 | 8.12 | 8.25 | 2.99 |
| Risk stillbirth | 0.08 | 19.88 | 20.15 | 20.42 | 5.04 | 5.10 | 5.17 | 7.98 | 8.11 | 8.24 | 3.01 |
| Risk stillbirth | 0.14 | 19.95 | 20.22 | 20.48 | 5.01 | 5.07 | 5.13 | 7.94 | 8.09 | 8.21 | 3.02 |
| Days low-shedding | 730 | 19.82 | 20.07 | 20.34 | 5.05 | 5.12 | 5.18 | 7.96 | 8.10 | 8.22 | 2.98 |
| Days low-shedding | 912 | 19.85 | 20.11 | 20.41 | 5.06 | 5.12 | 5.19 | 7.97 | 8.11 | 8.24 | 2.98 |
| Days low-shedding | 1095 | 19.90 | 20.12 | 20.39 | 5.07 | 5.13 | 5.19 | 7.99 | 8.12 | 8.25 | 2.99 |
| Days low-shedding | 1277 | 19.89 | 20.15 | 20.41 | 5.07 | 5.13 | 5.20 | 8.00 | 8.13 | 8.26 | 3.00 |
| Days low-shedding | 1460 | 19.89 | 20.15 | 20.41 | 5.07 | 5.14 | 5.20 | 8.01 | 8.13 | 8.27 | 3.00 |
| Days high-shedding | 182 | 19.86 | 20.10 | 20.37 | 5.06 | 5.12 | 5.19 | 7.97 | 8.11 | 8.24 | 2.99 |
| Days high-shedding | 365 | 19.90 | 20.12 | 20.39 | 5.07 | 5.13 | 5.19 | 7.99 | 8.12 | 8.25 | 2.99 |
| Days high-shedding | 548 | 19.87 | 20.13 | 20.37 | 5.07 | 5.13 | 5.19 | 7.98 | 8.13 | 8.24 | 3.00 |
| Days affected | 45 | 19.88 | 20.16 | 20.41 | 5.07 | 5.14 | 5.20 | 7.98 | 8.13 | 8.26 | 3.00 |
| Days affected | 91 | 19.92 | 20.16 | 20.44 | 5.08 | 5.13 | 5.19 | 8.00 | 8.13 | 8.26 | 3.00 |
| Days affected | 182 | 19.88 | 20.12 | 20.41 | 5.07 | 5.13 | 5.19 | 8.00 | 8.12 | 8.24 | 2.99 |
| Days affected | 225 | 19.85 | 20.11 | 20.42 | 5.06 | 5.12 | 5.19 | 7.99 | 8.12 | 8.26 | 3.00 |
| Days affected | 271 | 19.83 | 20.09 | 20.34 | 5.06 | 5.13 | 5.18 | 7.97 | 8.11 | 8.23 | 2.99 |
| Hygiene level | 1.2 | 19.89 | 20.15 | 20.39 | 5.07 | 5.14 | 5.20 | 8.00 | 8.13 | 8.27 | 2.99 |
| Hygiene level | 1.1 | 19.91 | 20.14 | 20.37 | 5.08 | 5.13 | 5.19 | 8.01 | 8.13 | 8.25 | 3.00 |
| Hygiene level | 1.0 | 19.90 | 20.12 | 20.39 | 5.07 | 5.13 | 5.19 | 7.99 | 8.12 | 8.25 | 2.99 |
| Hygiene level | 0.9 | 19.87 | 20.12 | 20.36 | 5.07 | 5.13 | 5.19 | 7.99 | 8.12 | 8.24 | 2.99 |
| Hygiene level | 0.8 | 19.84 | 20.07 | 20.37 | 5.06 | 5.12 | 5.19 | 7.96 | 8.10 | 8.24 | 2.98 |
| Hygiene level | 0.7 | 19.78 | 20.02 | 20.30 | 5.04 | 5.11 | 5.17 | 7.95 | 8.08 | 8.20 | 2.97 |
| Hygiene level | 0.6 | 19.70 | 19.97 | 20.25 | 5.03 | 5.10 | 5.16 | 7.91 | 8.06 | 8.19 | 2.96 |
| Hygiene level | 0.5 | 19.62 | 19.87 | 20.14 | 5.02 | 5.08 | 5.15 | 7.88 | 8.02 | 8.14 | 2.93 |
| Test specificity | 70 | 19.70 | 19.95 | 20.22 | 5.03 | 5.09 | 5.16 | 7.91 | 8.05 | 8.17 | 2.95 |
| Test specificity | 80 | 19.76 | 20.02 | 20.29 | 5.04 | 5.11 | 5.17 | 7.93 | 8.08 | 8.20 | 2.97 |
| Test specificity | 90 | 19.81 | 20.08 | 20.32 | 5.06 | 5.12 | 5.18 | 7.97 | 8.10 | 8.22 | 2.98 |
| Test specificity | 95 | 19.85 | 20.08 | 20.36 | 5.06 | 5.12 | 5.19 | 7.98 | 8.11 | 8.24 | 2.99 |
| Test specificity | 98.67 | 19.90 | 20.12 | 20.39 | 5.07 | 5.13 | 5.19 | 7.99 | 8.12 | 8.25 | 2.99 |
| Test specificity | 99 | 19.87 | 20.11 | 20.38 | 5.06 | 5.13 | 5.19 | 7.98 | 8.11 | 8.24 | 2.99 |
| Test specificity | 99.5 | 19.87 | 20.12 | 20.39 | 5.07 | 5.13 | 5.19 | 7.99 | 8.12 | 8.25 | 2.99 |
| Sensitivity level | 0.5 | 19.94 | 20.19 | 20.44 | 5.08 | 5.14 | 5.20 | 8.02 | 8.15 | 8.26 | 3.01 |
| Sensitivity level | 0.6 | 19.91 | 20.17 | 20.43 | 5.07 | 5.14 | 5.20 | 8.00 | 8.14 | 8.26 | 3.00 |
| Sensitivity level | 0.7 | 19.92 | 20.15 | 20.43 | 5.07 | 5.13 | 5.20 | 8.01 | 8.13 | 8.27 | 3.00 |
| Sensitivity level | 0.8 | 19.91 | 20.15 | 20.43 | 5.08 | 5.14 | 5.21 | 8.00 | 8.14 | 8.26 | 3.00 |
| Sensitivity level | 0.9 | 19.87 | 20.14 | 20.42 | 5.07 | 5.13 | 5.20 | 7.99 | 8.13 | 8.25 | 3.00 |
| Sensitivity level | 1 | 19.90 | 20.12 | 20.39 | 5.07 | 5.13 | 5.19 | 7.99 | 8.12 | 8.25 | 2.99 |
| Sensitivity level | 1.1 | 19.86 | 20.11 | 20.38 | 5.06 | 5.13 | 5.19 | 7.98 | 8.12 | 8.25 | 2.99 |
| Sensitivity level | 1.2 | 19.85 | 20.09 | 20.38 | 5.06 | 5.12 | 5.19 | 7.97 | 8.11 | 8.23 | 2.99 |

**Table 12.** Production and Economic results of the sensitivity analysis. Numbers are in million KG or million EUR. ECM = Milk yield, Exp = expenses, Inc = income, TNR = Total net revenue over ten years. Numbers describe the percentiles of 500 repetitions of 10 simulation years. Start prevalence = 5.6%. Prop. showing fast heat describes the proportion of cows that are not successfully inseminated and can be inseminated again after 21 days. Frac. low producing cows for cull.list describes the fraction of the lowest producing cows that the farmer flags for the culling list in the model. Days in calving box is the number of days spent in the calving box when calving. Feed unit cost is the cost of one feed unit. Wastemilk infection and colostrum risk are the daily risks of being infected from each source. Risk reduction when removing calves describes the reduction in risk of infection from the dam when the calf is removed within 2 hours after birth.

| Parameter | Value | ECM 5% | ECM 50% | ECM 95% | Exp. 5% | Exp. 50% | Exp. 95% | Inc. 5% | Inc. 50% | Inc. 95% | Median TNR |
| --- | --- | --- | --- | --- | --- | --- | --- | --- | --- | --- | --- |
| Prop. showing fast heat | 0.5 | 19.90 | 20.12 | 20.39 | 5.07 | 5.13 | 5.19 | 7.99 | 8.12 | 8.25 | 2.99 |
| Prop. showing fast heat | 0.1 | 19.90 | 20.12 | 20.39 | 5.07 | 5.13 | 5.19 | 7.99 | 8.12 | 8.25 | 2.99 |
| Prop. showing fast heat | 0.15 | 19.90 | 20.12 | 20.39 | 5.07 | 5.13 | 5.19 | 7.99 | 8.12 | 8.25 | 2.99 |
| Prop. showing fast heat | 0.25 | 19.90 | 20.12 | 20.39 | 5.07 | 5.13 | 5.19 | 7.99 | 8.12 | 8.25 | 2.99 |
| Prop. showing fast heat | 0.50 | 19.90 | 20.12 | 20.39 | 5.07 | 5.13 | 5.19 | 7.99 | 8.12 | 8.25 | 2.99 |
| Frac. low prod. cows for cull.list | 0.1 | 19.72 | 19.99 | 20.24 | 5.03 | 5.10 | 5.16 | 7.95 | 8.09 | 8.21 | 2.99 |
| Frac. low prod. cows for cull.list | 0.2 | 19.90 | 20.12 | 20.39 | 5.07 | 5.13 | 5.19 | 7.99 | 8.12 | 8.25 | 2.99 |
| Frac. low prod. cows for cull.list | 0.5 | 20.01 | 20.22 | 20.45 | 5.05 | 5.11 | 5.17 | 8.12 | 8.24 | 8.35 | 3.14 |
| Frac. low prod. cows for cull.list | 0.8 | 19.67 | 19.89 | 20.10 | 4.97 | 5.02 | 5.07 | 8.04 | 8.15 | 8.24 | 3.12 |
| Frac. low prod. cows for cull.list | 0 | 19.90 | 20.12 | 20.39 | 5.07 | 5.13 | 5.19 | 7.99 | 8.12 | 8.25 | 2.99 |
| Frac. low prod. cows for cull.list | 100 | 19.90 | 20.12 | 20.39 | 5.07 | 5.13 | 5.19 | 7.99 | 8.12 | 8.25 | 2.99 |
| Frac. low prod. cows for cull.list | 200 | 19.90 | 20.12 | 20.39 | 5.07 | 5.13 | 5.19 | 7.99 | 8.12 | 8.25 | 2.99 |
| Frac. low prod. cows for cull.list | 300 | 19.90 | 20.12 | 20.39 | 5.07 | 5.13 | 5.19 | 7.99 | 8.12 | 8.25 | 2.99 |
| Days in calving box | 1 | 19.90 | 20.12 | 20.39 | 5.07 | 5.13 | 5.19 | 7.99 | 8.12 | 8.25 | 2.99 |
| Days in calving box | 3 | 19.90 | 20.12 | 20.39 | 5.07 | 5.13 | 5.19 | 7.99 | 8.12 | 8.25 | 2.99 |
| Days in calving box | 5 | 19.90 | 20.12 | 20.39 | 5.07 | 5.13 | 5.19 | 7.99 | 8.12 | 8.25 | 2.99 |
| Days in calving box | 10 | 19.90 | 20.12 | 20.39 | 5.07 | 5.13 | 5.19 | 7.99 | 8.12 | 8.25 | 2.99 |
| Feed unit cost | 0.100 | 19.90 | 20.12 | 20.39 | 5.07 | 5.13 | 5.19 | 7.99 | 8.12 | 8.25 | 2.99 |
| Feed unit cost | 0.133 | 19.90 | 20.12 | 20.39 | 5.07 | 5.13 | 5.19 | 7.99 | 8.12 | 8.25 | 2.99 |
| Feed unit cost | 2.00 | 19.90 | 20.12 | 20.39 | 5.07 | 5.13 | 5.19 | 7.99 | 8.12 | 8.25 | 2.99 |
| Feed unit cost | 5.00 | 19.90 | 20.12 | 20.39 | 5.07 | 5.13 | 5.19 | 7.99 | 8.12 | 8.25 | 2.99 |
| Wastemilk infection risk | 0.0022 | 19.89 | 20.13 | 20.39 | 5.07 | 5.13 | 5.20 | 7.99 | 8.13 | 8.26 | 2.99 |
| Wastemilk infection risk | 0.0032 | 19.89 | 20.13 | 20.39 | 5.07 | 5.13 | 5.20 | 7.99 | 8.13 | 8.26 | 2.99 |
| Wastemilk infection risk | 0.0042 | 19.89 | 20.13 | 20.39 | 5.07 | 5.13 | 5.20 | 7.99 | 8.13 | 8.26 | 2.99 |
| Colostrum infection risk | 0.01 | 19.88 | 20.14 | 20.41 | 5.07 | 5.14 | 5.20 | 7.99 | 8.13 | 8.26 | 2.99 |
| Colostrum infection risk | 0.02 | 19.88 | 20.14 | 20.41 | 5.07 | 5.14 | 5.20 | 7.99 | 8.13 | 8.26 | 2.99 |
| Colostrum infection risk | 0.05 | 19.88 | 20.14 | 20.41 | 5.07 | 5.14 | 5.20 | 7.99 | 8.13 | 8.26 | 2.99 |
| Risk reduction when removing calves | 0.00 | 19.85 | 20.12 | 20.39 | 5.13 | 5.20 | 5.26 | 7.98 | 8.12 | 8.25 | 2.92 |
| Risk reduction when removing calves | 0.50 | 19.88 | 20.13 | 20.37 | 5.14 | 5.20 | 5.27 | 7.98 | 8.12 | 8.25 | 2.92 |
| Risk reduction when removing calves | 0.95 | 19.88 | 20.13 | 20.37 | 5.14 | 5.20 | 5.26 | 7.99 | 8.12 | 8.25 | 2.93 |
| Risk reduction when removing calves | 1.00 | 19.87 | 20.13 | 20.37 | 5.14 | 5.20 | 5.26 | 7.98 | 8.13 | 8.24 | 2.93 |

**Figure 6.** The variance of the median total net revenue estimate when resampling 100 to 1000 simulations. The plot shows 20 resamplings with different seed. From this analysis we deemed 500 repetitions to be adequate for the model to converge since the variation appears to have reached a stationary rather than a rapidly decreasing state.


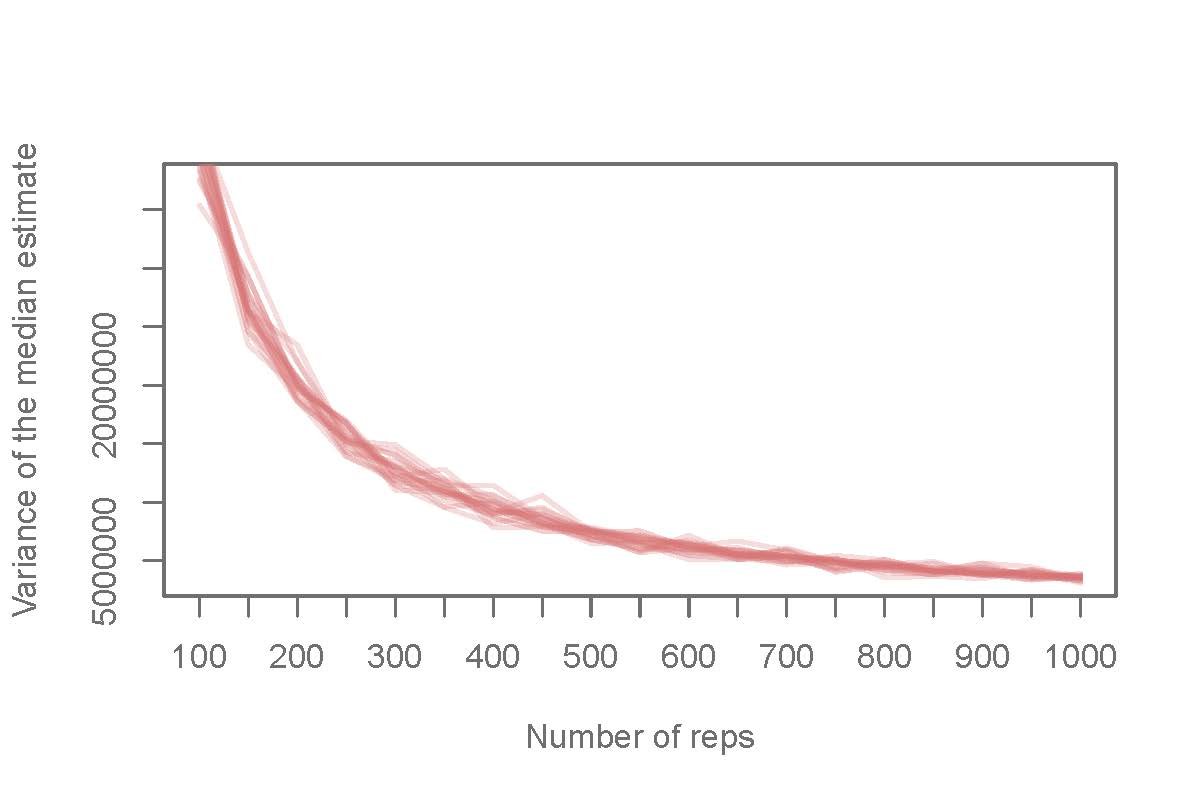


**Additional references used in the Supplementary Materials:**

Aes O. *Optimalt foderniveau til højtydende malkekøer*. Appendix from “Theme Day for Feeding Issues”. (2009). p. 1–7. Available from: www.landbrugsinfo.dk

Ancker S, Nørremark T, Fogh A, Sloth HK, Trinderup M. *Business Check Kvæg* 2013. Danish Cattle Federation (2009). p. 1–68. Available from: www. landbrugsinfo.dk
